# Comprehensive Flow Cytometry Profiling of the Immune System in COVID-19 Convalescent Individuals

**DOI:** 10.3389/fimmu.2021.793142

**Published:** 2022-01-06

**Authors:** Sergio Gil-Manso, Iria Miguens Blanco, Rocío López-Esteban, Diego Carbonell, Luis Andrés López-Fernández, Lori West, Rafael Correa-Rocha, Marjorie Pion

**Affiliations:** ^1^ Laboratory of Immune-Regulation, Gregorio Marañón Health Research Institute (IiSGM), Gregorio Marañón University General Hospital, Madrid, Spain; ^2^ Department of Emergency, Gregorio Marañón University General Hospital, Madrid, Spain; ^3^ Department of Hematology, Gregorio Marañón Health Research Institute (IiSGM), Gregorio Marañón University General Hospital, Madrid, Spain; ^4^ Service of Pharmacy, Gregorio Marañón Health Research Institute (IiSGM), Gregorio Marañón University General Hospital, Madrid, Spain; ^5^ Department of Pediatrics, Alberta Transplant Institute and Canadian Donation and Transplantation Research Program, University of Alberta, Edmonton, AB, Canada; ^6^ Department of Medical Microbiology & Immunology, Alberta Transplant Institute and Canadian Donation and Transplantation Research Program, University of Alberta, Edmonton, AB, Canada; ^7^ Department of Surgery, Alberta Transplant Institute and Canadian Donation and Transplantation Research Program, University of Alberta, Edmonton, AB, Canada; ^8^ Department of Laboratory Medicine & Pathology, Alberta Transplant Institute and Canadian Donation and Transplantation Research Program, University of Alberta, Edmonton, AB, Canada

**Keywords:** COVID-19, immune system, flow cytometry, unsupervised algorithms, immune dysregulation

## Abstract

SARS-CoV-2 has infected more than 200 million people worldwide, with more than 4 million associated deaths. Although more than 80% of infected people develop asymptomatic or mild COVID-19, SARS-CoV-2 can induce a profound dysregulation of the immune system. Therefore, it is important to investigate whether clinically recovered individuals present immune sequelae. The potential presence of a long-term dysregulation of the immune system could constitute a risk factor for re-infection and the development of other pathologies. Here, we performed a deep analysis of the immune system in 35 COVID-19 recovered individuals previously infected with SARS-CoV-2 compared to 16 healthy donors, by flow cytometry. Samples from COVID-19 individuals were analysed from 12 days to 305 days post-infection. We observed that, 10 months post-infection, recovered COVID-19 patients presented alterations in the values of some T-cell, B-cell, and innate cell subsets compared to healthy controls. Moreover, we found in recovered COVID-19 individuals increased levels of circulating follicular helper type 1 (cTfh1), plasmablast/plasma cells, and follicular dendritic cells (foDC), which could indicate that the Tfh-B-foDC axis might be functional to produce specific immunoglobulins 10 months post-infection. The presence of this axis and the immune system alterations could constitute prognosis markers and could play an important role in potential re-infection or the presence of long-term symptoms in some individuals.

## Introduction

Up to now, the COVID-19 pandemic has affected more than 230 million people and has claimed the lives of more than 4.8 million people worldwide. COVID-19 is induced by the Severe acute respiratory syndrome coronavirus 2 (SARS-CoV-2). Infected individuals range from asymptomatic to presenting with severe symptoms, with a median fatality rate of 0.27% (1). After infection, the immune system manages to control it successfully in most cases, generating an immunological memory. More than 80% of infected people are asymptomatic or develop mild symptoms (2). However, some of them suffer from long-term COVID-19-associated symptoms after the infection is resolved (3). In some cases, the virus triggers an exacerbated immune response that goes from protecting to attacking the infected individual. During the inflammatory response, an increase in pro-inflammatory cytokines, T cell activation, and T cell exhaustion was observed (4–6). At the same time, decreases in regulatory cells, T-cell cytotoxicity, and T cells’ polyfunctionality were observed (5, 7–9). Even when deeper dysregulation is linked to severe disease, it was observed that COVID-19 individuals, even with mild symptoms, also present immune dysregulation (10).

Due to the interest in the possible acquisition of strong immune protection after natural infection, numerous studies have analysed the immune-specific response against SARS-CoV-2 in convalescent individuals. However, the impact of the infection on the whole immune system after recovery has not been studied. As a result of increasing evidence of long-term COVID-19 symptoms after viral clearance (11–13), there is growing interest in understanding whether immunologic dysregulation may persist among convalescent individuals versus uninfected healthy individuals. With more than 230 million COVID-19 cases documented worldwide, the long-term COVID-19 individual numbers are growing every day, and therefore, the health consequences of SARS-CoV-2 infection and their subsequent socioeconomic costs are far beyond those of active infection alone.

Therefore, a deep understanding of the state of the immune system after natural infection could give important information about the duration of immune dysregulation or the immune response to possible re-infection. Moreover, knowing the immune status after infection, even in individuals who no longer present symptoms, is necessary to determine the risks and the sequelae that may remain.

We performed a deep analysis of innate and adaptive immune cells in 35 COVID-19 convalescent individuals with previous asymptomatic/mild symptoms and 16 non-infected individuals. Our study revealed that various cellular subsets associated with innate or adaptive compartments were differentially expressed between the groups 10 months post-infection. More importantly, some of them could be pivotal to fight future re-infection. These results provide important insights into the potential immune consequences that can mark the future health of previously infected individuals.

## Materials and Methods

### Patients and Blood Samples

Blood samples and data questionnaires of donor characteristics during COVID-19 from SARS-CoV-2 convalescent donors were collected from June to December 2020, and healthy controls were collected from January to February 2021, at the General University Hospital Gregorio Marañón, Spain. Informed consent was obtained under the Declaration of Helsinki protocol. The study was approved and performed according to local ethics committees (COV1-20-007). SARS-CoV-2 infection was confirmed by PCR test after nasopharyngeal swab. SARS-CoV-2 donor recruitment was conducted in healthcare workers in the General University Hospital Gregorio Marañón in Madrid, infected with SARS-CoV-2 between March and December 2020. Sample collection was performed at a single time point, between 12 days post-positive PCR (P-PCR+) and 305 days P-PCR+. Detailed healthy and recovered individuals’ characteristics are provided in **Table 1**.

**Table 1:** Demographic and clinical characteristics and comorbidities in healthy and recovered individuals.

Characteristics	Healthy control (n = 16)	Recovered COVID19 (n = 35)	p-value
**Age (years), median (range)**	43,5 (23-59)	40 (25-62)	0.805
**Gender, n (%)**			0.753
Male	6 (37.5)	11 (31.4)	–
Female	10 (62.5)	24 (68.6)	–
**Ethnicity, n (%)**			0.543
Caucasian	16 (100)	32 (91.43)	–
Latin American	0 (0.0)	3 (8.57)	–
**Comorbidities, n (%)**			
Current smoker/ex-smoker	2 (12.5)/3 (18.75)	3 (8.6)/4 (11.4)	0.671
Asthma	1 (6.25)	3 (8.6)	1.000
Obesity	1 (6.25)	1 (2.9)	1.000
Allergy	1 (6.25)	0 (0.0)	0.314
Heart disease	0 (0.0)	2 (5.7)	0.561
Hypertension	0 (0.0)	1 (2.9)	1.000
Epilepsy	0 (0.0)	1 (2.9)	1.000
Psoriasis	0 (0.0)	1 (2.9)	1.000
Sleep apnea	0 (0.0)	1 (2.9)	1.000
Fibromyalgia	0 (0.0)	1 (2.9)	1.000
Diabetes	0 (0.0)	0 (0.0)	–
Kidney disease	0 (0.0)	0 (0.0)	–
Liver disease	0 (0.0)	0 (0.0)	–
**Symptoms during COVID-19, n (%)**			
Fatigue	–	19 (54.3)	
Myalgia	–	19 (54.3)	
Anosmia	–	16 (45.7)	
Fever (≥38)	–	14 (40.0)	
Headache	–	14 (40.0)	
Ageusia	–	13 (37.1)	
Cough	–	13 (37.1)	
Diarrhea	–	10 (28.6)	
Dyspnea	–	9 (25.7)	
Arthralgia	–	5 (14.3)	
Nausea or vomiting	–	5 (14.3)	
Fever (<38)	–	3 (8.6)	
Pneumonia	–	3 (8.6)	
Dizziness	–	3 (8.6)	
Tachycardia	–	3 (8.6)	
Sore throat	–	2 (5.7)	
Conjunctivitis	–	1 (2.9)	
Congestion	–	1 (2.9)	
Skin rash	–	1 (2.9)	

Characteristics of the healthy controls (n = 16) and recovered COVID-19 patients (COV, n = 35). The total number of individuals is indicated for all the characteristics and symptoms, except for age (years). A Mann–Whitney U test was performed to analyse age differences between groups. Fisher’s exact test was performed to analyze the rest of the characteristics.

### Cell Surface Marker Staining

Whole blood was labelled for surface markers with the antibodies and their fluorochromes distributed in four flow cytometry panels named T-cell, B-cell, Tfh–Tγδ cell, and innate immune cell panels (**Table S1**). CD80 and CD86 are used with the same fluorochrome in the aim to detect the activated B cells. After surface labelling, red blood cells were lysed using RBC Lysis/Fixation Solution (Bio-Legend, San Diego, CA, USA). Surface markers were analysed by flow cytometry using a MACSQuant Analyser 16 cytometer (Miltenyi Biotec, Bergisch Gladbach, Germany). Whole blood was labelled within 2 h of the extraction.

### Detection of Cytokine Levels in Plasma

Cytokine levels were measured in plasma samples employing the automated immunoassay ELLA (Protein Simple, San Jose, CA, USA). We used two different simple plex panels to study the levels of IL-1 β, IL-6, IL-8, TNF- α, CCL2, IL-10, CXCL10, GM-CSF, and IFNγ. The determination of cytokine levels was done using Simple Plex Runner v. 3.7.2.0 software (San Jose, CA, USA). If any measurement was below or above the detection range, we set the minimum or maximum detection limit as value.

### Unsupervised Analysis of the Four Flow Cytometry Panels

In addition to doing traditional manual gating from cytometry data, as presented in the **Supplementary Materials**, we performed a high-dimensional flow cytometric analysis in the four flow cytometry panels using three different algorithms in Cytobank (www.cytobank.org): viSNE, FlowSOM, and CITRUS. viSNE (visualisation of t-distributed Stochastic Neighbour Embedding) is an algorithm that reduces high-parameter data down to two dimensions and allows for easy visualisation of all markers in each cytometry panel and detects visual differences in specific cell subsets. We used the following settings: 1,300,000 events were analysed under proportional sampling between the individuals from total events. Iteration: 7,000; perplexity: 30; theta: 0.5 with a random seed. Onto the viSNE reduced dimension, we ran FlowSOM clustering (Self-Organizing Map from Flow cytometry). FlowSOM is another algorithm to transform cell clusters into higher-order metaclusters. We selected this algorithm because it reveals cell subsets that could be overlooked when using classical manual gating. FlowSOM settings randomly selected 13 individuals in the COV group and the CT group, and the sampling was done with equal event numbers between individuals. Clustering method: hierarchical consensus; number of metaclusters: 15; number of clusters: 100; iterations: 100 with a random seed. CITRUS (cluster identification, characterisation, and regression) was the third algorithm used and is designed for fully automated discovery of statistically significant stratifying biological signatures.

As for the analysis of FlowSOM, we randomly selected 13 individuals from the COV group and the CT group, and the sampling was done with equal event numbers between individuals. We ran two predictive association models: (i) the Nearest Shrunken Centroid (PAMR) and (ii) the L1-Penalized Regression (LASSO *via* GLMNET). Cluster characterization: abundance, event sampling: equal; minimum cluster size: 1%; cross-validation Folds: 13; false discovery rate; 1%. For the T-cells panel, the unsupervised analyses were done on CD3+ T-cells. For the B-cells panel, the unsupervised analyses were done on CD19/CD20 gated B-cells. For the Tfh–Tγδ cells panel, the unsupervised analyses were done on CD3+ gated T-cells. For the innate immune cell panel, the unsupervised analyses were done on gated leukocytes.

### Titration for SARS-CoV-2 Antibodies Using Luminex Single-Antigen Beads

SARS-CoV-2 S1 (Abcam) and RBD (Sino Biological, Wayne, PA, US) proteins were conjugated to Luminex beads using standard coupling procedures (14). Coupling was confirmed using a rabbit IgG anti-SARS-CoV-2 Spike monoclonal antibody (Sino Biological) and PE-conjugated goat anti-rabbit IgG secondary antibody (Southern Biotech). To detect SARS-CoV-2 antibodies, sera (25-fold dilution) were incubated with Luminex beads for 30 min at room temperature, washed, and then incubated with a 50-fold dilution of secondary antibody for 30 min at room temperature. Samples were acquired using a FLEXMAP 3D^®^ Luminex system (Toronto, Canada). Cut-off for SARS-CoV-2 S1 and RBD was 1000 MFI and 5000 MFI, respectively.

### Software and Statistical Analysis

Flow cytometry data was analysed using Kaluza version 2.1 and Cytobank algorithms (both from Beckman Coulter, Brea, CA, USA). Data from flow cytometry is displayed as the mean with standard error deviation (SEM). Data from the medians of fluorescent intensity (MFI) is displayed as the median with SEM. A description of the statistical tests used to evaluate the experiments is provided within the respective figure legends. Continuous data was tested for distribution, and individual groups were tested using the Mann–Whitney U test. Spearman’s rho (r) was calculated for the correlation between continuous data. *P*-value significance levels were corrected using the Benjamini–Hochberg method for multiple testing. Adjusted *p*-values of <0.05 were considered statistically significant. Graphs were plotted using GraphPad Prism 7.0. Statistical analyses were conducted using GraphPad Prism 7.0 (GraphPad, San Diego, CA, USA) and SPSS (IBM, version 25, Armonk, NY, USA) software.

## Results

### COVID-19 and Healthy Control Cohorts

We recruited 35 PCR-confirmed COVID-19 individuals; 4 were asymptomatic, 29 presented with mild symptoms, and 2 presented with moderate symptoms according to the WHO classification (15) (**Table 1**). They were healthcare workers at General University Hospital Gregorio Marañón in Madrid, infected by SARS-CoV-2 between March and December 2020. Recovered subjects (COV) provided a blood sample at a single time point, between 12 days post-PCR (P-PCR+) and 305 days P-PCR+. Ninety-four percent of subjects were never hospitalised for COVID-19; 6% were hospitalised (*n* = 2), but none required intensive care unit (ICU) care. Sixteen healthy individuals were recruited and assessed as controls (CT). CT individuals never presented COVID-19 symptoms and were negative for anti-SARS-CoV-2 antibodies at the time of the sample extraction. No difference in comorbidities between the groups was observed (**Table 1**).

### Residual Plasmatic Inflammation Observed in Recovery Individuals

**Figure 1:**
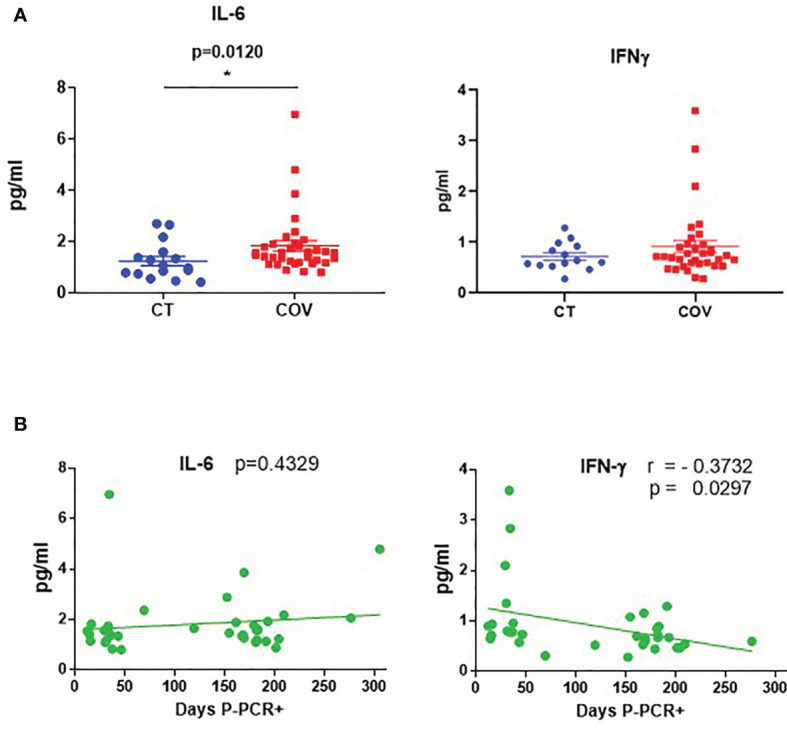
Cytokine levels in recovered COVID-19 and healthy individuals. **(A)** Cytokine levels of IL-6 and IFN-γ in healthy (CT) and recovered individuals (COV). Mean ± SEM. Pairwise comparisons were performed by a Mann–Whitney U-test corrected using the Benjamini–Hochberg method for multiple testing. **(B)** Correlation between days P-PCR+ and IL-6 and IFN-γ levels. A linear regression curve is represented in each graph. Correlations were done using Spearman’s rank-order correlation test; r = Spearman’s rank correlation coefficient. *P* = *p*-value, adjusted by the Benjamini–Hochberg adjustment method for multiple testing. *p < 0.05. Each symbol corresponds to an individual.

We measured a wide range of pro-inflammatory cytokines in plasma samples related to COVID-19 infection in the infected individuals at the time of the samples extraction. We did not find any differences in cytokines (IL-1β, IL-8, TNF-α, CCL2, IL-10, CXCL10, GM-CSF, and IFN-γ) between recovered and healthy individuals, except for IL-6 levels (**Figure 1A**). Recovered individuals showed slightly higher IL-6 mean levels than those of healthy controls (1.83 ± 0.203 pg/mL; 1.20 ± 0.19 pg/mL, respectively; *p* = 0.012). Because samples from recovered patients were analysed 12 to 305 days post-PCR+ (P-PCR+), we also investigated possible changes in cytokine levels as time passed. We observed no correlation between days P-PCR+ and IL-6 levels (*p* = 0.4329) and one negative correlation between days P-PCR+ and IFN-γ (r = –0.3732, *p* = 0.0297, **Figure 1B**). This negative correlation might indicate that, the longer ago the infection was, the less IFN-γ patients have in their plasma, reaching a basal non-inflammatory level of this cytokine (1 pg/mL).

### Activation of T-Cell Subsets in Recovered COVID-19 Individuals

**Figure 2:**
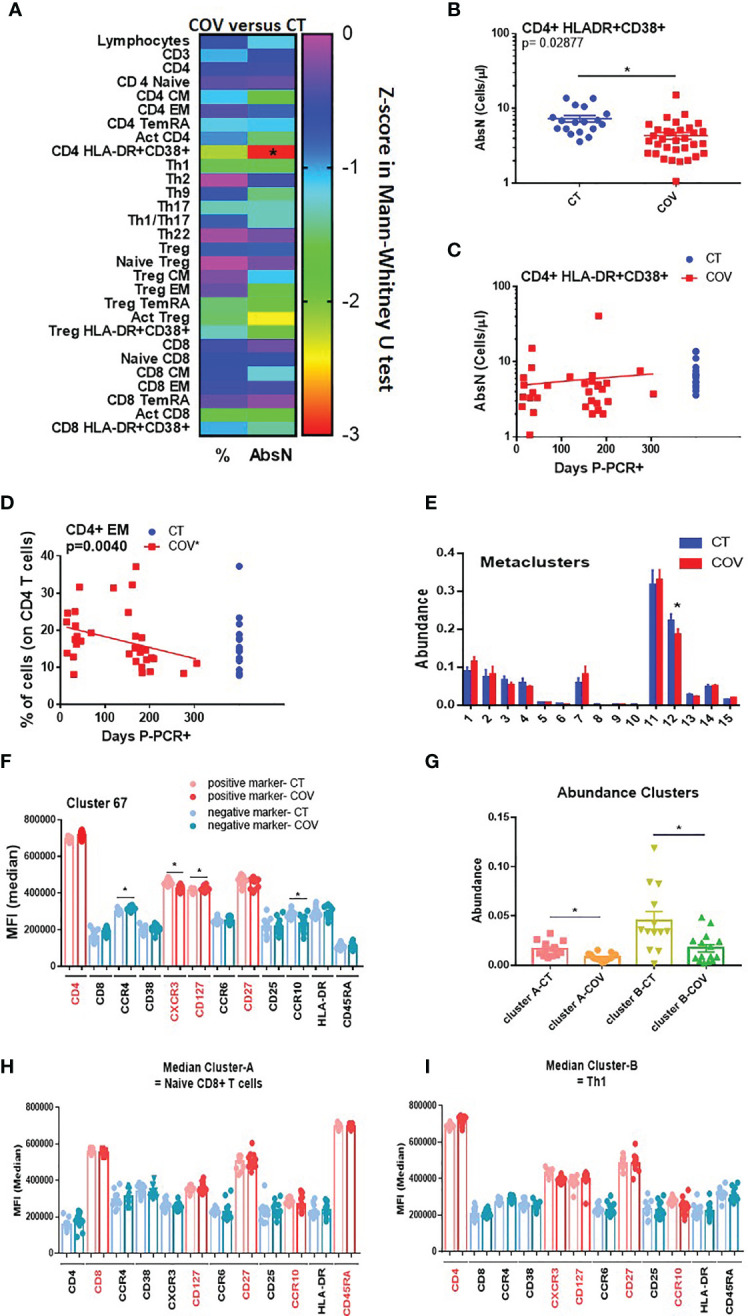
Manual gating and high-dimensional flow cytometry unsupervised analysis in T-cell panel. **(A)** Heat map of the pairwise comparison between recovered COVID-19 (COV) and healthy control (CT) individuals of results obtained by classical manual flow cytometry gating. Statistical analysis was performed with the Mann–Whitney U test. The colour scale represents the Z-score on the right Y-axis. Immune population names are represented on the left Y-axis. The left column represents the z-score from the pairwise comparison for the cellular population’s percentage (%), and the right column represents the z-score from the pairwise comparison for the absolute numbers (cells/uL, AbsN). The *p*-value was corrected using the Benjamini–Hochberg method for multiple testing. **(B)** AbsN of CD4+ HLA-DR+ CD38+ in CT and COV individuals. Pairwise comparisons were performed using a Mann–Whitney U-test corrected using the Benjamini–Hochberg method for multiple testing; mean ± SEM. **(C)** Correlation between days P-PCR+ and CD4+ HLA-DR+ CD38+ AbsN. Spearman’s rank-order correlation test with Benjamini–Hochberg adjustment for multiple testing. **(D)** Correlation between days P-PCR+ and frequency of CD4+ effector memory (EM). Spearman’s rank-order correlation test with Benjamini–Hochberg adjustment for multiple testing. **(E)** The metaclusters’ abundance was obtained through FlowSOM analysis. Two-way ANOVA with Benjamini–Hochberg adjustment for multiple testing. Median ± SEM. **(F)** The median of fluorescence (MFI) of cluster 67 was obtained through a FlowSOM analysis. One-way ANOVA with Benjamini–Hochberg adjustment for multiple testing. Median ± SEM. **(G)** The clusters’ abundance was significantly different between COV and CT individuals obtained through CITRUS analysis. One-way ANOVA with Benjamini–Hochberg adjustment for multiple testing. Median ± SEM. **(H)** The median of fluorescence (MFI) of cluster A or cluster B **(I)** was obtained through CITRUS analysis. One-way ANOVA with Benjamini–Hochberg adjustment for multiple testing. Median ± SEM. *p < 0.05.

We studied T-cell subsets, using traditional manual gating (**Figure S1**), and found that the absolute number of the activated CD4+ HLA-DR+ CD38+ T cells subset was significantly different between the groups (**Figure 2A** and **Figure S1**), being lower in the COV group than in the CT group (**Figure 2B**; CD4+ HLA-DR+ CD38+ T cells 4.38 ± 0.412 cells/µL and 6.82 ± 0.748 cells/µL, absolute number mean ± SEM, respectively, in the COV and CT groups).

Because the samples were extracted from 12 to 305 days P-PCR+, we investigated possible changes in subsets regarding the time P-PCR+. No significant correlation was observed in terms of the distribution of the absolute numbers of CD4+ HLA-DR+ CD38+ as time passed (**Figure 2C**). We also observed that, even if no difference was seen between the groups, the frequency of CD4 effector memory (EM) decreased significantly as time passed from infection (**Figure 2D**), indicating a diminution in the frequency of differentiated CD4+ T cells.

We then applied a high-dimensional flow cytometry analysis to explore lymphocyte activation and differentiation between recovered COVID-19 and healthy individuals. Using the unsupervised algorithms (viSNE), we detected only a few variations in the distribution of cellular populations between the CT and COV groups (data not shown). Using the viSNE results, we ran a Self-Organising Map from flow cytometry (FlowSOM), which permits clustering cells that can reveal how all markers are behaving in all cells. All 35 recovered COVID-19 individuals were analysed independently of the time post-infection. From the 15 metaclusters generated, one showed a significant difference in abundance between the groups (metacluster 12, **Figure 2E**), being more abundant in the CT group than in the COV group. Metacluster 12 was composed of 22 clusters (**Figure S2A**), but only one of them (cluster 67, **Figure S2B**) was significantly different between the COV and CT groups (**Figure S2C**). We observed that the phenotype of this metacluster was CD4+ CD45RAneg CCR4neg CCR10neg CD27+ CCR6neg CXCR3+ CD127+ (**Figure 2F** and **Figure S2D**), which corresponds to the effector Th1 central memory subset. This subset presented a significantly lower mean of fluorescence intensity for the CXCR3 marker in the COV group than in the CT group; as well as a trafficking marker that promotes Th1 response, and CCR10, a skin-homing marker (**Figure 2F**).

After the viSNE analysis, we ran the CITRUS algorithm (cluster identification, characterisation, and regression), which is designed for the automated discovery of statistically significant biological signatures within datasets (CT versus COV). Two clusters were discovered to have higher abundance in the CT than in the COV group (**Figure 2G**). Regarding the fluorescence intensity of each panel’s markers, the first cluster (**Figure 2H**—cluster A, and **Figure S3**) was defined as CD8+ CD127+ CD27+ CCR10+ CD45RA+, which may correspond to the naïve CD8+ T cell subset. The second cluster (**Figure 2I**—cluster B) was defined as CD4+ CXCR3+ CCR6neg CCR4neg CD127+ CD27+ CCR10+ (**Figure S3**), related to the Th1 central memory, confirming the results obtained by the FlowSOM analysis. Surprisingly, both subsets expressed the CCR10 marker that is generally associated with skin or mucosal-resident T-cells (16, 17). This marker is generally not associated with Th1 or naïve CD8+ T cells.

In summary, recovered COVID-19 individuals presented sustained lower counts of activated CD4+ T cells than healthy controls. The unsupervised analyses permitted us to detect that CT group individuals presented a higher abundance of Th1 central memory and naïve CD8+ T cells, both expressing the mucosal homing receptor CCR10. This diminution is likely due to residual lymphopenia, but it cannot be ruled out that these cells expressing CCR10 could also be still present in tissues instead of recirculating in the periphery in convalescent individuals.

### The Type-1 T Follicular Helper Subset Is More Frequent in Recovered Than in Healthy Individuals

**Figure 3:**
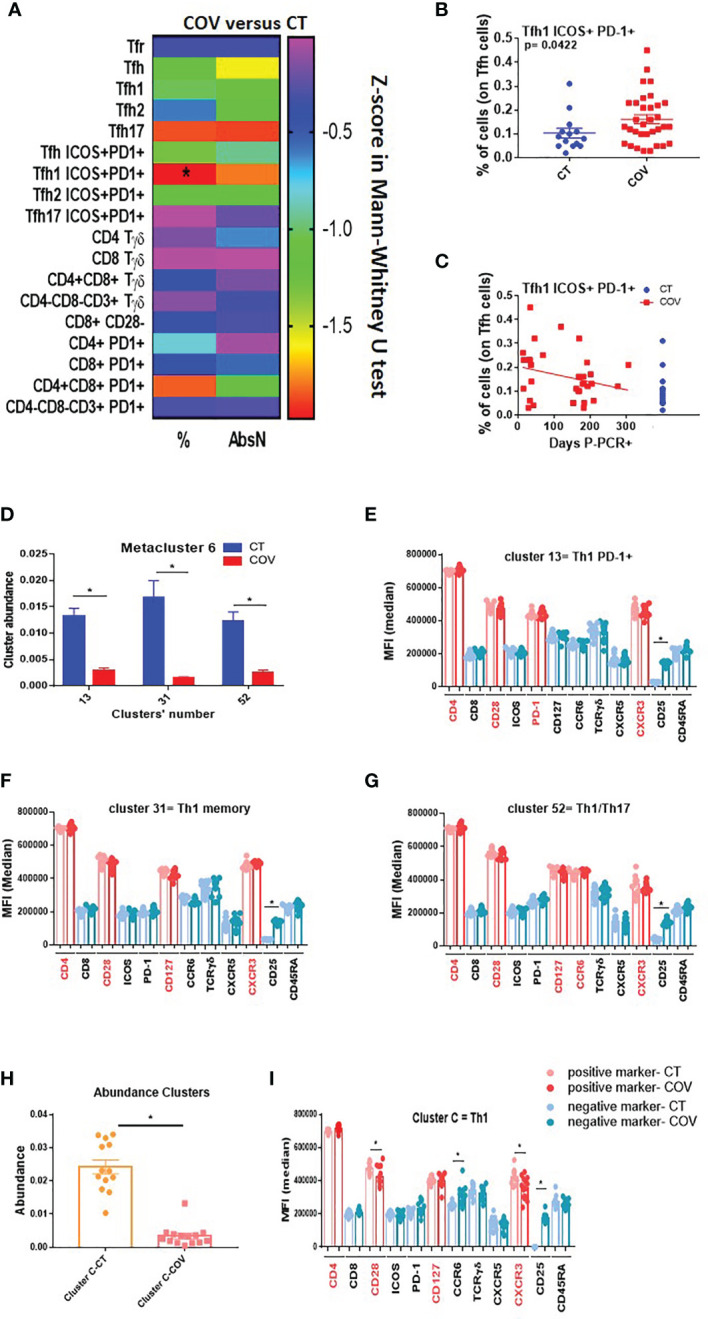
Manual gating and high-dimensional flow cytometry unsupervised analysis in Tfh–Tγδ cells panel. **(A)** Heat map of the pairwise comparison between recovered COVID-19 (COV) and healthy control (CT) individuals of cellular subsets obtained by classical manual flow cytometry gating. Analysis was performed with the Mann–Whitney U test. The colour scale represents Z-score on the right Y-axis. Immune population names are represented on the left Y-axis. The left column represents the z-score from the pairwise comparison for the cellular population’s percentage (%), and the right column represents the z-score from the pairwise comparison for the absolute numbers (cells/uL, AbsN). p-value was adjusted by the Benjamini–Hochberg adjustment method for multiple testing, *p < 0.05. **(B)** Frequency of Tfh1 ICOS+ PD-1+ in CT and COV individuals. Pairwise comparisons were performed using a Man–Whitney U-test with Benjamini–Hochberg adjustment for multiple testing. Mean ± SEM. **(C)** Correlation between days P-PCR+ and frequency of Tfh ICOS+ PD-1+. Spearman’s rank-order correlation test with Benjamini–Hochberg adjustment for multiple testing. **(D)** The abundance of the three metaclusters was obtained through FlowSOM analysis. One-way ANOVA with Benjamini–Hochberg adjustment for multiple testing. Median ± SEM. **(E)** Medians of fluorescence (MFI) of clusters 13, 31 **(F)**, and 52 **(G)** were obtained through FlowSOM analysis. One-way ANOVA with Benjamini–Hochberg adjustment for multiple testing. Median ± SEM. **(H)** The abundance of the cluster was significantly different between CT and COV individuals, as obtained through CITRUS analysis. One-way ANOVA with Benjamini–Hochberg adjustment for multiple testing. Median ± SEM. **(I)** MFI of cluster C was obtained through CITRUS analysis. One-way ANOVA with Benjamini–Hochberg adjustment for multiple testing. Median ± SEM. *p < 0.05.

Functional T cells such as pro-inflammatory and senescent T cells were also analysed. Using the traditional manual gating strategy (**Figure S4A**), we observed that the frequency of the peripheral or circular T follicular helper type-1 subset (cTfh1 ICOS+ PD-1+) was significantly higher in the COV group than in the CT group (**Figures 3A, B**). Moreover, even if not significant, the frequency of the cTfh1 ICOS+ PD-1+ subset was higher in individuals with early infection than in individuals with a longer time post-infection (**Figure 3C**).

The FlowSOM algorithm was run on a viSNE analysis, and one metacluster (metacluster 6) was significantly more represented in the CT than in the COV group (**Figure S4B**), with a clear expression of CD4 and no expression of CD25 (**Figure S4C**). This metacluster comprises three clusters (clusters 13, 31, and 52, **Figure S5A**) and was significantly more abundant in the CT group than in the COV group (**Figure 3D**). Cluster 13 expressed CD4+ CD28+ CXCR3+ PD-1+, which could be related to a Th1 PD-1+ subset (**Figure 3E** and **Figure S5B**). Cluster 31 expressed CD4+ CD28+ CD45RAneg CD127+ CXCR3+, which could be related to the memory Th1 subset (**Figure 3F** and **Figure S5B**), and the cluster 52 expressing CD4+ CD28+ CD127+ CCR6+ CXCR3+ could be related to the memory Th1/Th17 subsets (**Figure 3G** and **Figure S5B**).

To confirm these results, we used the second clustering algorithm, CITRUS, which permitted us to discover statistically significant biological signatures between COV and CT. One cluster was significantly less represented in the COV group (**Figure 3H**, cluster C). Cluster C was related to the Th1 memory subset and the expression of CD4+ CD28+ CD127+ CXCR3+ (**Figure 3I** and **Figure S6**), confirming the previous discovery by FlowSOM analysis (**Figure 3F**). Moreover, the MFI of CD28 and CXCR3 were diminished in the COV group compared to the CT group (**Figure 3I**). Therefore, the difference between the groups was due, not only to the cell abundance, but also to the markers’ expression intensity.

Summing up, we confirmed in this panel that Th1 and Th1/Th17 were differentially represented in both groups, with greater abundance in the CT than in the COV group, likely due to remnant lymphopenia. Furthermore, COV individuals presented higher frequencies of the activated cTfh1 subset (ICOS+ PD-1+) in the COV group than in the CT group, independent of the sampling time, which are implicated in the B-cell response during the infection.

### B Cell Activation in Recovered COVID-19 Individuals

**Figure 4:**
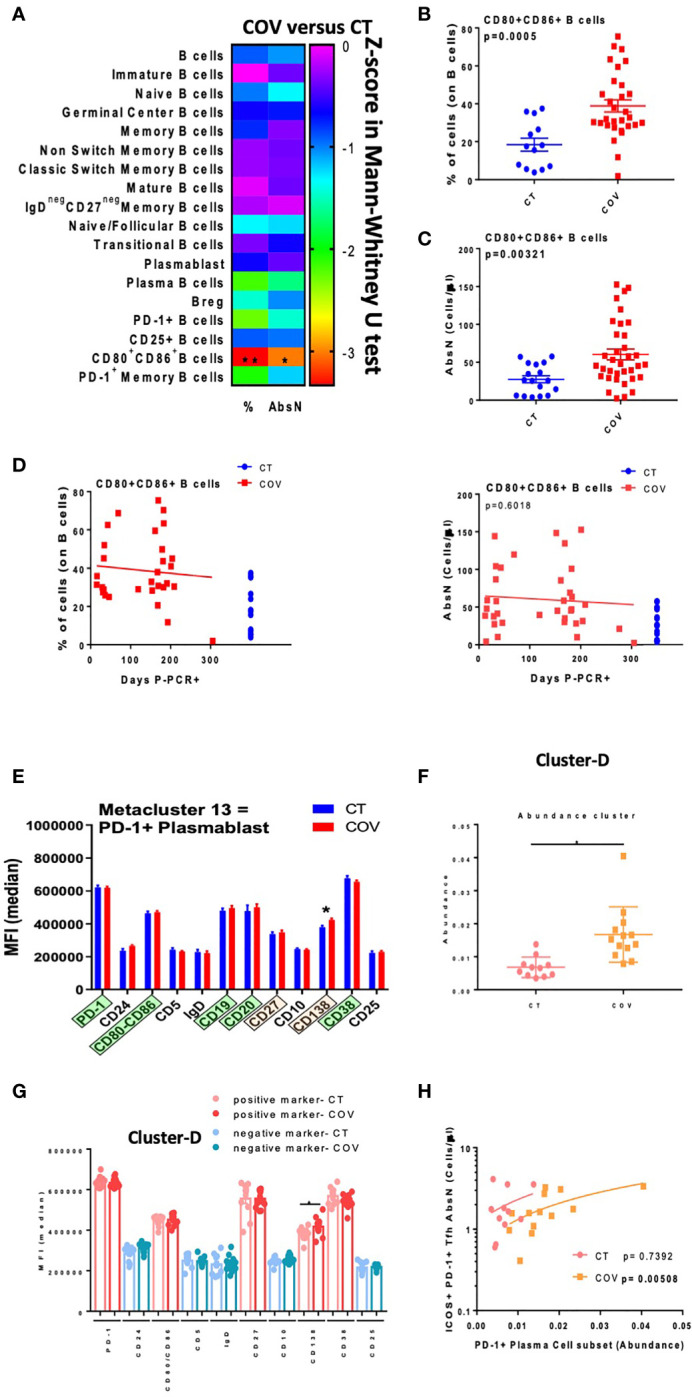
Manual gating and high-dimensional flow cytometry unsupervised analysis in B-cell panel. **(A)** Heat map of the pairwise comparison between recovered COVID-19 (COV) and healthy control (CT) individuals of cellular subsets obtained by classical flow cytometry analysis. Analysis was performed with the Mann–Whitney U test. The colour scale represents the Z-score on the right Y-axis. Immune population names are represented on the left Y-axis. The left column represents the z-score from the pairwise comparison of the cellular population’s percentage (%), and the right column represents the z-score from the pairwise comparison of the absolute numbers (cells/uL, AbsN). The *p*-value was adjusted by the Benjamini–Hochberg adjustment method for multiple testing. **(B)** Frequency or AbsN **(C)** of CD80/CD86+ B-cells in CT and COV individuals. Pairwise comparisons were performed using a Mann–Whitney U-test with Benjamini–Hochberg adjustment for multiple testing. Mean ± SEM. **(D)** Correlation between days P-PCR+ and frequency of CD80/CD86+ (left panel) and AbsN of CD80/CD86+ B cells (right panel). Spearman’s rank-order correlation test with Benjamini–Hochberg adjustment for multiple testing. **(E)** MFI of cluster 13 was obtained through FlowSOM analysis. One-way ANOVA with Benjamini–Hochberg adjustment for multiple testing. Median ± SEM. **(F)** The abundance of cluster D was significantly different between COV and CT individuals and was obtained through CITRUS analysis. One-way ANOVA with Benjamini–Hochberg adjustment for multiple testing. Median ± SEM. **(G)** MFI of cluster D obtained through CITRUS analysis. One-way ANOVA with Benjamini–Hochberg adjustment for multiple testing. Median ± SEM. **(H)** Correlation between AbsN of ICOS+ PD-1+ Tfh and the abundance of PD-1+ plasma cells. Spearman’s rank-order correlation test with Benjamini–Hochberg adjustment for multiple testing. *p < 0.05 and **p < 0.01.

cTfh1 is related to B cell response and immunoglobulin secretion; therefore, we analysed the B-cell differentiation and activation phenotypes using a classical gating strategy (**Figure S7**). A significant difference in the frequency and absolute numbers of activated B cells (CD80/CD86+, **Figure 4A**) was observed, with a higher frequency (**Figure 4B**) and AbsN (**Figure 4C**) in the COV group than in the CT group. However, the frequencies and AbsN of CD80+ CD86+ B cells (**Figure 4D**, right panel) were not correlated with the sampling time, showing that the higher frequencies and AbsN of activated B cells persist. In future studies, it will be essential to discriminate CD80+ and CD86+ B cells, and not only the combination of CD80/CD86 since CD80 and CD86 are not only activation markers, but they might also be differentially expressed on B cells. Therefore, CD80 and CD86 markers can represent B cells with different function.

The unsupervised FlowSOM analysis permitted us to detect one metacluster (metacluster 13) with a significantly higher abundance in the COV group than in the CT group, even if this cluster represented a minority subset (**Figure S8A**). Metacluster 13 was related to PD-1+-expressing plasmablasts since it presented CD19+ CD20+ CD80/CD86+ CD38+ markers (**Figure 4E** and **Figure S8B**). It was already described that pre-plasmablasts and plasmablasts could express CD80 and CD86 (18). Because of the intermediary expression of CD138 in these cells, one can assume that they were plasmablasts differentiating into plasma cells. Moreover, in the CITRUS algorithm applied to the two groups of individuals, only one cluster was defined as predictively different between CT and COV, with a higher abundance in COV than in the CT group (**Figure 4F**). This cluster was expressing CD80/CD86+ CD27+ CD38+ CD138+ PD-1+ in the surface of the cells—all markers that could be related to PD-1+ plasma cells (**Figure 4G** and **Figure S9**). Moreover, the CD138 MFI was significantly higher in the COV group than in the CT group (417,290 ± 11,410 and 382,224 ± 9505, MFI ± SEM, respectively, *p* = 0.0479). Therefore, from two different unsupervised analyses, we found that individuals in the COV group presented more PD-1+ plasmablasts and PD-1+ plasma cells than in the CT group, showing that immunoglobulin-producing cells were present in recovered individuals. Interestingly, the abundance of the PD-1+ plasma cell subset was found to be positively correlated with absolute counts of cTfh ICOS+ PD-1+ (p= 0.00508) in the COV group but not in the CT group (p= 0.7392, **Figure 4H**). In summary, COV group individuals presented a sustained activated B-cell compartment with higher abundance of PD-1+ plasma cells and plasmablast subsets than healthy controls, likely due to a remnant of the viral infection. Antigen-activated B cells interact with follicular helper T cells to produce strong anti-antigen-specific immunoglobulins, and the ability of B cells to produce anti-SARS-CoV-2 specific immunoglobulins is essential to fight viral infection. Indeed, we observed that the abundance of PD-1+ plasma cells was correlated with the numbers of ICOS+ PD-1+ Tfh, which could evidence that the COVID-19 recovered individuals still have a solid Tfh-B cell axis 10 months post-infection.

### Innate Immunity in Recovered COVID-19 Individuals

**Figure 5:**
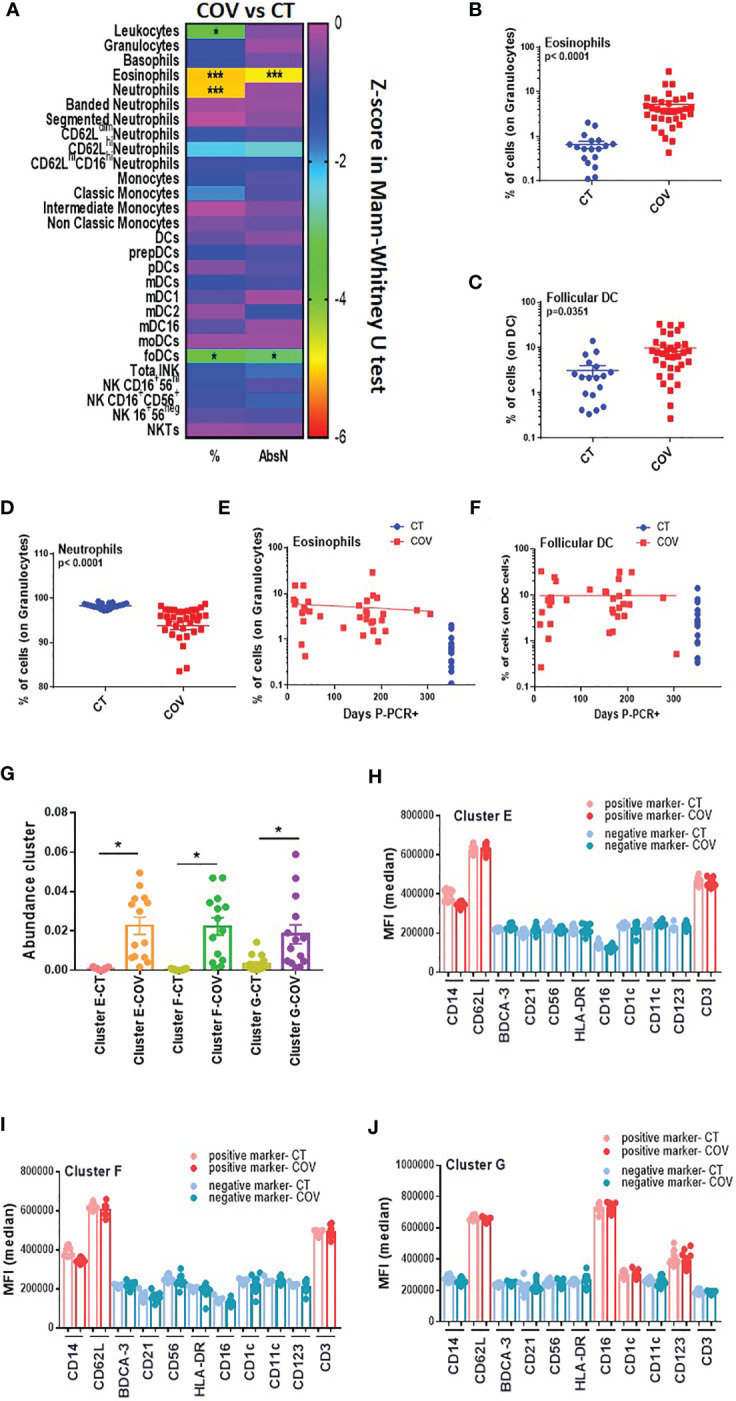
Manual gating and high-dimensional flow cytometry unsupervised analysis in innate cells panel. **(A)** Heat map of the pairwise comparison between recovered COVID-19 (COV) and healthy control (CT) individuals of cellular subsets obtained by classical flow cytometry analysis. The analysis was performed with the Mann–Whitney U test. The colour scale represents the Z-score on the right Y-axis. Immune population names are represented on the left Y-axis. The left column represents the z-score from the pairwise comparison of the cellular population’s percentage (%), and the right column represents the z-score from the pairwise comparison of the absolute numbers (cells/uL, AbsN). The *p*-value was adjusted by the Benjamini–Hochberg adjustment method for multiple testing. *p < 0.05, and ***p < 0.001. **(B)** Frequency of eosinophils and **(C)** follicular DC in CT and COV individuals. Pairwise comparisons were performed by Mann–Whitney U-test with Benjamini–Hochberg adjustment for multiple testing. Mean ± SEM. **(D)** Frequency of neutrophils in CT and COV individuals. Pairwise comparisons were performed using Mann–Whitney U-test with Benjamini–Hochberg adjustment for multiple testing. Mean ± SEM. **(E)** Correlation between days P-PCR+ and frequency of eosinophils or **(F)** follicular DC. Spearman’s rank-order correlation test with Benjamini–Hochberg adjustment for multiple testing. **(G)** The abundance of clusters was significantly different between CT and COV individuals and was obtained through CITRUS analysis. One-way ANOVA with Benjamini–Hochberg adjustment for multiple testing. Median ± SEM. **(H)** MFI of cluster E, **(I)** cluster F, and **(J)** cluster G were obtained through CITRUS analysis. One-way ANOVA with Benjamini–Hochberg adjustment for multiple testing. Median ± SEM. *p < 0.05.

Innate immunity is also crucial for developing a solid immune response, and patients with mild symptoms also presented dysregulation of innate immunity (19). Using the traditional manual gating strategy (**Figure S10**), we detected a significant difference in frequencies and AbsN for several cellular subsets, such as eosinophils, neutrophils, and follicular DCs (foDCs) (**Figure 5A**), with an increased frequency of eosinophils and foDC in the COV group compared to the CT group (**Figures 5B, C**), but a decreased frequency of neutrophils in the COV group in comparison to the CT group (**Figure 5D**). At 10 months post-infection, there were no correlations between eosinophils and foDC frequencies and time P-PCR+ and both subsets showed sustained high frequencies as time passed post-infection (**Figures 5E, F**).

The CITRUS analysis detected three clusters that were significantly more abundant in the COV group than in the CT group (**Figure 5G**). Clusters E and F (**Figures 5H, I**) presented almost the same phenotype, CD14+ CD3+ CD62L+, representing an unconventional CD14+ CD3+ double-positive subset that was already associated with immune dysregulation (20) (**Figure S11**). We did not determine CD14+ CD3+ doublet since our analysis was done in the singlet gate, and therefore, we cannot conclude whether this double positive subset is a real subset expressing both markers or if it was composed by T-cell: monocyte complexes, as observed in the ref 20.

Cluster G expressed HLA-DRneg CD11cneg CD14neg CD62L+ CD16+ CD123+ CD1cint (**Figure 5J**). While CD16 and CD1c are markers for myeloid dendritic cells (CD1c+ mDC and CD16+ mDC), CD123 is a marker for plasmacytoid dendritic cells. More surprisingly, HLA-DR and CD11c were not expressed in this cluster. Both markers are generally used during the first step for the total DC gating strategy. Therefore, this cluster could also represent an atypical DC subset that has not been detected by manual gating.

In summary, this panel demonstrated that innate immune dysregulation was still observed 10 months post-infection with atypical DC subsets associated with recovered COVID-19 individuals.

**Figure 6:**
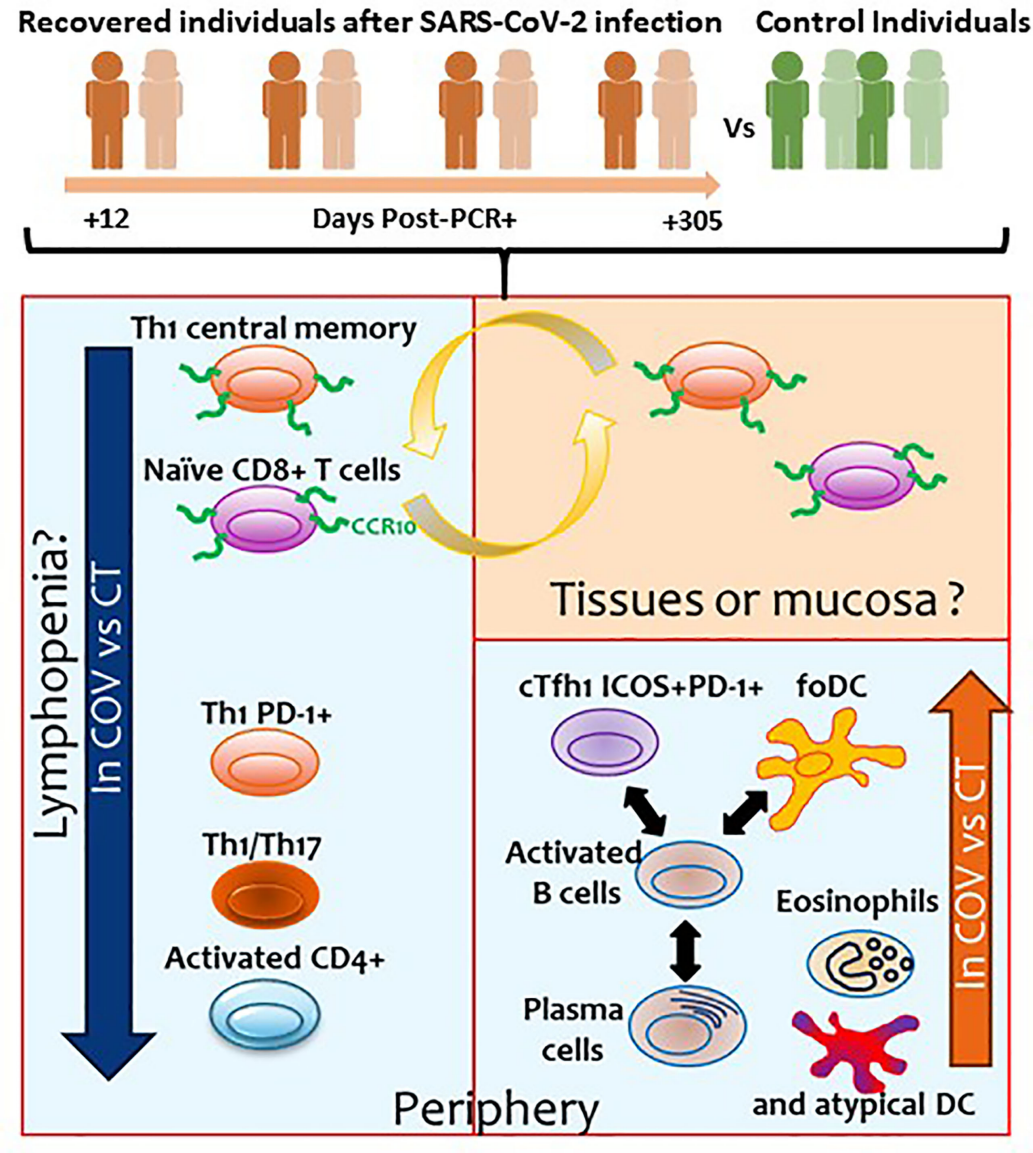
Summary of the results obtained in the present study. Orange and green individuals represent the recovered individuals after SARS-CoV-2 infection and controls, respectively. Numbers represents the days post-PCR+ when the samples of the former COVID-19 individuals were analysed. Blue squares represent the cellular subsets with altered levels observed in the periphery. Orange square represents the cellular subsets that could be potentially found in tissues or mucosa.

The results of the four cytometry panels are recapitulated in **Figure 6**.

## Discussion

The great majority of COVID-19 individuals present mild symptoms or are asymptomatic, but little is known about the status of the immune system in COVID-19 individuals after asymptomatic/mild disease. In this study, we performed comprehensive immune profiling in COVID-19 recovered patients using a traditional gating strategy and different unsupervised algorithms. We compared the results with healthy individuals with no SARS-CoV-2 antecedent to determine possible immune subsets dysregulated due to past infection. The detection and identification of these subsets could help us better understand the immune system after SARS-CoV-2 infection and determine which individuals could be prone to reinfection. In addition, this study can help us understand the long-term symptoms that some recovered COVID-19 individuals may suffer. The results are summarised in **Table 2** and **Figure 6**.

**Table 2:** Summary of the principal cellular subsets significantly and differentially abundant between COV and CT group individuals.

Inferior in COV-Group compared to CT-group	
Cellular subsets	Frequency/AbsN
CD4+ HLADR+ CD38+ → **activated CD4+ T cells**	AbsN
CD4+ CD45RAneg CCR4neg CCR10neg CCR6neg CD27+ CXCR3+ CD127+ → **Th1 central memory**	Frequency
CD8+ CD127+ CD27+ CCR10+ CD45RA+ → **atypical naïve CD8**	Frequency
CD4+ CXCR3+ CD127+ CD27+ CCR10+ → **atypical Th1 central memory**	Frequency
CD4+ CD28+ CXCR3+ PD-1+ → **Tfh1 PD-1+**	Frequency
CD4+ CD28+ CD45RAneg CD127+ CXCR3+ → **memory Th1**	Frequency
CD4+ CD28+ CD45RAneg CD127+ CXCR3+ CCR6+ → **memory Th1/Th17**	Frequency
**Neutrophils**	Frequency
**Superior in COV-Group compared to CT-group**	
**Cellular subsets**	**Frequency/AbsN**
**Tfh1** ICOS+ **PD-1+** → **cTfh1 ICOS+ PD-1+**	Frequency
CD80+/CD86+ B cells → **activated B cells**	Frequency/AbsN
CD80/CD86+ CD38+ CD27int CD138int B cells → **plasmablasts**	Frequency
CD80/CD86+ CD27+ CD138+ CD38+ PD-1+ B cells → **PD-1+ plasma cells**	Frequency
**Eosinophils**	Frequency/AbsN
**Follicular DC**	Frequency/AbsN
CD14+ CD3+ CD62L+ → **unconventional double positive**	Frequency
CD3neg CD14neg CD62L+ CD16+ CD123+ CD1c int → **atypical DC**	Frequency

At the top of the table are the cell subsets with a frequency or AbsN lower in the COV group compared to the CT group, while the bottom part of the table has the subsets with a frequency or AbsN greater in the COV group compared to the CT group. The left column gives the cellular subsets and their most representative markers. The right column indicates if a difference between groups was observed in terms of subsets’ frequency or absolute numbers.

We found only a few dysregulated immune cell subsets in recovered patients compared to healthy controls. Some of them were atypical subsets that could be key to understanding the infection, such as the double-positive CD14+CD3+ subset observed within the ‘live singlet’ events gate, a T-cell/monocyte complex described in diseases where the immune system is disturbed (20). Indeed, Burel JG. et al. have also demonstrated that the T-cell/monocyte complexes are observed in the living singlet gate. These complexes are formed due to an increase of adhesion molecules at their surface leading to a higher constant of association between both T (cells) and monocyte subsets. These complexes were observed essentially during the acute phase of active tuberculosis or acute dengue fever infection (20). Acute tuberculosis and dengue fever present similarities with SARS-CoV-2-associated symptoms (21, 22) and the three pathogens were also able to increase peripheral cytokines’ levels such as IFN-γ and thus, dysregulate the innate and adaptive immune system (23, 24). Even though we have not determined if the CD14+ CD3+ subset observed in our study is related to those complexes, there are associated with former COVID-19 individuals who presented immune system inflammation and dysregulation. Therefore, this CD14+ CD3+ subset could surge from the activation of the immune system during SARS-CoV-2 infection, but its role in the disease progression or viral clearance is not known and further studies will be needed to determine their possible implication in reinfection protection.

It was also observed an atypical DC subset characterised by the low HLA-DR and CD11c expression, intermediate expression of CD1c and high expression of CD16 and CD123. CD123 is a general marker for plasmacytoid DC, and CD1c or CD16 are markers for myeloid DC. Therefore, this subset presents some DC characteristics, but it has not yet been described to our knowledge. A rare DC subset named CD16+ slanDC presenting CD14neg CD1c+ with high CD16 expression and low expression of HLA-DR in their immature form has already been observed (25–27). However, we cannot determine if this subset could be related to immature CD16+ slanDC since the expression of CD123 on these cells was not described. Nevertheless, it was reported that precursor myeloid cells could express CD123 (28, 29). Therefore, one can hypothesise that the atypical DC subset determined in our study was related to a precursor or an immature state of CD16+ DC. This subset was depicted to be a pro-inflammatory DC subset (30) and could explain why they are found in recovered COVID-19 instead of healthy individuals.

The role of the CD3+ CD14+ and atypical DC subsets is unknown, and we cannot conclude that these subsets are a consequence of the inflammation after SARS-CoV-2 infection or if they could have helped during the viral clearance. Therefore, further studies will be needed to determine their possible implication in reinfection, protection or disease severity.

As expected, diminished frequencies and absolute counts of leukocytes, naïve, activated, and effector (Th1 or Th17) CD4+ T cells can be associated with a remnant of lymphopenia already observed in the majority of COVID-19 individuals (9, 10, 31) and recovered individuals (32). However, the lower abundance in the COV group compared to the CT group of the atypical Th1 memory and atypical naïve CD8 T-cells, both expressing CCR10, could have one other explanation. Indeed, CCR10 is a skin- and mucosal-homing marker (16, 17). Therefore, one can assume that these subsets can still be found in the airways, mucosa, and/or inflamed tissues in recovered individuals. Indeed, SARS-CoV-2 infects the epithelial airways, and local inflammation occurs. It was shown that SARS-CoV-2 ORF7 could induce the expression of CCL27, one of the CCR10 ligands (33). Moreover, CCL27 and CCL28 serum levels are high during SARS-CoV-2 infection (34–36) and were shown to be upregulated in the lungs during the late stages of SARS infection (37). COVID-19 individuals often have lung and other organ damage where high concentrations of the CCR10 ligands have been described (38, 39). Therefore, one can suppose that CCL27 and/or CCL28 could be expressed in the lung and that CCR10-expressing cells could be attracted to the inflammatory site, diminishing their frequency in the periphery.

Another key observation is that after 10 months post-infection, the frequency and absolute counts of activated B cells (CD80+/CD86+) were higher in convalescent individuals. CD80 and CD86 are two markers expressed on naïve B cells upon stimulation. In this study, these markers were labelled with the same fluorochrome to determine such activation. Therefore, it was not possible to distinguish between CD80+-B cells and CD86+-B cells. Further studies will be needed to distinguish both B-cell subsets into COVID-19 individuals since it was demonstrated that both could have differential functions in different pathologies (40–42). Indeed, CD80 was associated with pro-inflammatory cytokine stimulation, while CD86 could play a protective role mediated through anti-inflammatory cytokines in APC. More importantly, CD86 was highly expressed after type-I-IFN stimulation in the marginal zone of the lymph node where they could promote autoimmune response and participate in the co-stimulation of CD4 T cells (43). Therefore, the level of CD80+- and CD86+-B cells in recovered COVID-19 should be studied to determine if those cells have a role in protecting the individuals from reinfection.

In the total B cell subset, PD-1+ plasmablasts and plasma cells were more abundant in recovered COVID-19 individuals than in healthy controls. Plasmablasts are the precursor subset of plasma cells. They are recognisable for their ability to secrete large numbers of antibodies. An increase in the number of atypical memory B cells and plasma cells had already been observed in COVID-19 individuals (44). In our work, the immunoglobulin-producing subsets expressed the immunomodulatory markers PD-1+ at high levels. PD-1 was described as a negative regulator of B-cell activation (45). Indeed, a diminution of anti-SARS-CoV-2 and neutralising antibodies had already been observed over time in convalescent individuals, even though a potential long-lasting humoral B-cell memory subset was detected (32, 46–48). Therefore, it is not clear if these PD-1+ plasmablasts/plasma cells could produce a sustained level of anti-SARS-CoV-2 immunoglobulins. Further studies are necessary to elucidate the protective role of PD-1+ plasma cells in in the long term.

FoDC are non-migratory DC subtypes and are generally found in the secondary lymph nodes. The formation of the functional GC requires an architecture composed of different sorts of leukocytes, especially foDC (49). FoDC intervenes in specific B-cell response generation after forming the germinal center (GC), where the B cells are differentiated into plasma cells to produce protective high-affinity antibodies (50). Circulating foDC have been described in patients with chronic hepatitis B virus infection (51), and their frequencies positively correlate with plasma cells; foDC could contribute to the efficient immune responses against the pathogen. In this work, we also found higher circulating foDC frequencies in the peripheral blood of recovered COVID-19 individuals compared to healthy controls. Tfh are also essential for germinal centre formation, as well as in regulation and B cell differentiation into plasma cell producers of high-affinity antibodies. The expression of ICOS and PD-1 points to activated cTfh cells and plays an essential role in regulating germinal centre formation, B-cell survival, and B-cell differentiation into long-lived plasma cells (52). It is already described that after SARS-CoV-2 infection, there is a production of S-specific antibodies, memory B cells and cTfh cells (53). Here, we show that the absolute numbers of cTfh ICOS+ PD-1+ are positively correlated with the abundance of the PD-1+ plasma subset, as already described (54, 55). ICOS and PD-1 expression in cTfh is reported to be increased in several immune-related diseases, such as ulcerative colitis (56) and multiple sclerosis (57), or associated with disease severity in such conditions as Primary Sjogren’s Syndrome (58). Thus, the ICOS+PD-1+ cTfh subset presence in recovered individuals could be related to past inflammation during infection. Also, cTfh cells have been related to the production of neutralising antibody titers in COVID-19 convalescent individuals (59), which may indicate that the durability of the antibody titers is due to the cTfh cells, among others. It was already observed that anti-SARS-CoV-2-S IgG titers persist for 12 months (60, 61) along with cTfh cells for at least 6 months after SARS-CoV-2 infection (62). The fact that absolute numbers of this subset are correlated with the abundance of the PD-1+ plasma cell subset in recovered COVID-19 individuals, could indicate that the past-inflammation was related to a plasma B cell response in individuals who were presenting mild/moderate symptoms and thus raises hope for long-lasting COVID-19 immunity.

Therefore, besides PD-1+ plasma cells and activated B-cells, the presence of sustained high frequencies or absolute counts of cTfh1 ICOS+PD-1+ and circulating foDC could also be explained by the destructuring of the germinal centre in the lymphoid organs due to inflammation, as already observed during fatal COVID-19 (63, 64). In our work, we study individuals with asymptomatic/mild COVID-19; thus, it is unlikely that these individuals will present a deficiency in germinal centre organisation. Therefore, their presence is likely due to sustain residual activation of the immune system, which could be the hallmark of a solid foDC-Tfh-B cells axis at 10 months post-infection, which could effectively produce specific anti-SARS-CoV-2 antibodies after reactivation. Consequently, one can hypothesise that these patients would be protected from possible reinfection, as already proposed (65, 66).

It would be interesting to understand the function of these rare population (double-positive CD3+ CD14+, CCR10-Th1/CCR10-CD8+ T cells and atypical DC), and perform functional assays or deep sequencing to study their implication in convalescent individuals after SARS-CoV-2 infection. However, this is a limitation of this study since more than 95% of the health workers have been vaccinated, therefore the recruitment of the volunteers with or without previous infection is challenging. Indeed, we cannot affirm that these subsets have not been altered or are even present in those vaccinated individuals. Since most healthcare workers are vaccinated, another limitation of the study is the number of individuals analysed, and the difficulty to recruit more individuals to strengthen the findings of this work. Therefore, further studies are urgently needed to determine the exact role of circulating foDC and Tfh during and after SARS-CoV-2 infection, and the assessment of the presence of GC and foDC in lymphoid organs is highly desirable since GC formation is critical for long-lived memory or high-affinity B cells.

## Data Availability Statement

The original contributions presented in the study are included in the article/**Supplementary Material**. Further inquiries can be directed to the corresponding author.

## Ethics Statement

The studies involving human participants were reviewed and approved by Gregorio Marañón ethics committee (REF: COV1-20-007). The patients/participants provided their written informed consent to participate in this study.

## Author Contributions

Conceptualisation: MP. Data curation: MP, SG-M, DC, and RL-E. Formal Analysis: MP and SG-M. Funding acquisition: MP and RC-R. Investigation: MP, SG-M, DC, and RL-E. Methodology: MP and SG-M. Project administration: MP. Resources: MP, IM-B, LL-F, and LW. Supervision: MP. Validation: MP, LW, SG-M, LL-F, and IM-B. Writing, original draft: MP and SG-M. Review and editing: LL-F, RC-R, IM-B, DC, and RL-E revised the manuscript. All the authors interpreted and discussed the data. All the authors read and approved the final manuscript.

## Funding

This work was partially financed by the Madrid Community grant B2017/BMD3727 and the IiSGM Intramural grant PI-MP-2018. This work was partially funded by a grant from “Fundación Familia Alonso” (FFA-FIBHGM-2019). SG-M was supported by the Youth Employment Program, co-financed by the Madrid community and FEDER Founds (PEJ-2020-AI/BMD-17954), and by the ACT4COVID consortium (CellNex funding). The funders had no role in study design, data collection and analysis, decision to publish, or manuscript preparation. This work was partially supported by grants from the Instituto de Salud Carlos III (ISCIII) (PI18/00506; COV20/00063), co-funded by ERDF (FEDER) Funds from the European Commission, “A way of making Europe”.

## Conflict of Interest

The authors declare that the research was conducted in the absence of any commercial or financial relationships that could be construed as a potential conflict of interest.

## Publisher’s Note

All claims expressed in this article are solely those of the authors and do not necessarily represent those of their affiliated organizations, or those of the publisher, the editors and the reviewers. Any product that may be evaluated in this article, or claim that may be made by its manufacturer, is not guaranteed or endorsed by the publisher.

## References

[1] Ioannidis JPA. Infection Fatality Rate of COVID-19 Inferred From Seroprevalence Data. Bull World Health Organ (2021) 99(1):19–33F. doi: 10.2471/BLT.20.265892 33716331 PMC7947934

[2] Wang Y Chen Y Qin Q. Unique Epidemiological and Clinical Features of the Emerging 2019 Novel Coronavirus Pneumonia (COVID-19) Implicate Special Control Measures. J Med Virol (2020) 92(6):568–76. doi: 10.1002/jmv.25748 PMC7228347 32134116

[3] Akbarialiabad H Taghrir MH Abdollahi A Ghahramani N Kumar M Paydar S. Long COVID, A Comprehensive Systematic Scoping Review. Infection (2021) 49(6):1163–86. doi: 10.1007/s15010-021-01666-x PMC8317481 34319569

[4] Zheng HY Zhang M Yang CX Zhang N Wang XC Yang XP. Elevated Exhaustion Levels and Reduced Functional Diversity of T Cells in Peripheral Blood may Predict Severe Progression in COVID-19 Patients. Cell Mol Immunol (2020) 17(5):541–3. doi: 10.1038/s41423-020-0401-3 PMC7091621 32203186

[5] Zheng M Gao Y Wang G Song G Liu S Sun D. Functional Exhaustion of Antiviral Lymphocytes in COVID-19 Patients. Cell Mol Immunol (2020) 17(5):533–5. doi: 10.1038/s41423-020-0402-2 PMC7091858 32203188

[6] Thevarajan I Nguyen THO Koutsakos M Druce J Caly L van de Sandt CE. Breadth of Concomitant Immune Responses Prior to Patient Recovery: A Case Report of Non-Severe COVID-19. Nat Med (2020) 26(4):453–5. doi: 10.1038/s41591-020-0819-2 PMC7095036 32284614

[7] Qin C Zhou L Hu Z Zhang S Yang S Tao Y. Dysregulation of Immune Response in Patients With Coronavirus 2019 (COVID-19) in Wuhan, China. Clin Infect Dis (2020) 71(15):762–8. doi: 10.1093/cid/ciaa248 PMC7108125 32161940

[8] Laing AG Lorenc A Del Molino Del Barrio I Das A Fish M Monin L. A Dynamic COVID-19 Immune Signature Includes Associations With Poor Prognosis. Nat Med (2020) 26(10):1623–35. doi: 10.1038/s41591-020-1038-6 32807934

[9] Chen G Wu D Guo W Cao Y Huang D Wang H. Clinical and Immunological Features of Severe and Moderate Coronavirus Disease 2019. J Clin Invest (2020) 130(5):2620–9. doi: 10.1172/JCI137244 PMC7190990 32217835

[10] Mathew D Giles JR Baxter AE Oldridge DA Greenplate AR Wu JE. Deep Immune Profiling of COVID-19 Patients Reveals Distinct Immunotypes With Therapeutic Implications. Science (2020) 369(6508):eabc8511. doi: 10.1126/science.abc8511 32669297 PMC7402624

[11] Logue JK Franko NM McCulloch DJ McDonald D Magedson A Wolf CR. Sequelae in Adults at 6 Months After COVID-19 Infection. JAMA Netw Open (2021) 4(2):e210830. doi: 10.1001/jamanetworkopen.2021.0830 33606031 PMC7896197

[12] Del Rio C Collins LF Malani P. Long-Term Health Consequences of COVID-19. JAMA (2020) 324(17):1723–4. doi: 10.1001/jama.2020.19719 PMC8019677 33031513

[13] Garrigues E Janvier P Kherabi Y Le Bot A Hamon A Gouze H. Post-Discharge Persistent Symptoms and Health-Related Quality of Life After Hospitalization for COVID-19. J Infect (2020) 81(6):e4–6. doi: 10.1016/j.jinf.2020.08.029 PMC7445491 32853602

[14] Stephen A Shubhagata D Sherry D Valerie S Sarah S. XMAP Cookbook: A Collection of Methods and Protocols for Developing Multiplex Assays With xMAP Technolog. Austin, editor. Austin: Corporation L. TX2018 (2014).

[15] infection WWGotCCaMoC. A Minimal Common Outcome Measure Set for COVID-19 Clinical Research. Lancet Infect Dis (2020) 20(8):e192–e7. doi: 10.1016/S1473-3099(20)30483-7 PMC7292605 32539990

[16] Homey B Wang W Soto H Buchanan ME Wiesenborn A Catron D. Cutting Edge: The Orphan Chemokine Receptor G Protein-Coupled Receptor-2 (GPR-2, CCR10) Binds the Skin-Associated Chemokine CCL27 (CTACK/ALP/ILC). J Immunol (2000) 164(7):3465–70. doi: 10.4049/jimmunol.164.7.3465 10725697

[17] Pan J Kunkel EJ Gosslar U Lazarus N Langdon P Broadwell K. A Novel Chemokine Ligand for CCR10 and CCR3 Expressed by Epithelial Cells in Mucosal Tissues. J Immunol (2000) 165(6):2943–9. doi: 10.4049/jimmunol.165.6.2943 10975800

[18] Henn AD Laski M Yang H Welle S Qiu X Miao H. Functionally Distinct Subpopulations of CpG-Activated Memory B Cells. Sci Rep (2012) 2:345. doi: 10.1038/srep00345 22468229 PMC3315693

[19] Schultze JL Aschenbrenner AC. COVID-19 and the Human Innate Immune System. Cell (2021) 184(7):1671–92. doi: 10.1016/j.cell.2021.02.029 PMC7885626 33743212

[20] Burel JG Pomaznoy M Lindestam Arlehamn CS Weiskopf D da Silva Antunes R Jung Y. Circulating T Cell-Monocyte Complexes are Markers of Immune Perturbations. Elife (2019) 8:e46045. doi: 10.7554/eLife.46045 31237234 PMC6592685

[21] Thein TL Ang LW Young BE Chen MI Leo YS Lye DCB. Differentiating Coronavirus Disease 2019 (COVID-19) From Influenza and Dengue. Sci Rep (2021) 11(1):19713. doi: 10.1038/s41598-021-99027-z 34611200 PMC8492678

[22] Visca D Ong CWM Tiberi S Centis R D’Ambrosio L Chen B. Tuberculosis and COVID-19 Interaction: A Review of Biological, Clinical and Public Health Effects. Pulmonology (2021) 27(2):151–65. doi: 10.1016/j.pulmoe.2020.12.012 PMC7825946 33547029

[23] Cliff JM Kaufmann SH McShane H van Helden P O’Garra A. The Human Immune Response to Tuberculosis and Its Treatment: A View From the Blood. Immunol Rev (2015) 264(1):88–102. doi: 10.1111/imr.12269 25703554 PMC4368415

[24] Zheng W Wu H Liu C Yan Q Wang T Wu P. Identification of COVID-19 and Dengue Host Factor Interaction Networks Based on Integrative Bioinformatics Analyses. Front Immunol (2021) 12:707287. doi: 10.3389/fimmu.2021.707287 34394108 PMC8356054

[25] Rhodes JW Tong O Harman AN Turville SG. Human Dendritic Cell Subsets, Ontogeny, and Impact on HIV Infection. Front Immunol (2019) 10:1088. doi: 10.3389/fimmu.2019.01088 31156637 PMC6532592

[26] van Leeuwen-Kerkhoff N Lundberg K Westers TM Kordasti S Bontkes HJ de Gruijl TD. Transcriptional Profiling Reveals Functional Dichotomy Between Human Slan. J Leukoc Biol (2017) 102(4):1055–68. doi: 10.1189/jlb.3MA0117-037R 28720687

[27] Döbel T Kunze A Babatz J Tränkner K Ludwig A Schmitz M. Fcγriii (CD16) Equips Immature 6-Sulfo LacNAc-Expressing Dendritic Cells (slanDCs) With a Unique Capacity to Handle IgG-Complexed Antigens. Blood (2013) 121(18):3609–18. doi: 10.1182/blood-2012-08-447045 23460612

[28] Villani AC Satija R Reynolds G Sarkizova S Shekhar K Fletcher J. Single-Cell RNA-Seq Reveals New Types of Human Blood Dendritic Cells, Monocytes, and Progenitors. Science (2017) 356(6335):eaah4573. doi: 10.1126/science.aah4573 28428369 PMC5775029

[29] Collin M Bigley V. Human Dendritic Cell Subsets: An Update. Immunology (2018) 154(1):3–20. doi: 10.1111/imm.12888 29313948 PMC5904714

[30] Schäkel K Kannagi R Kniep B Goto Y Mitsuoka C Zwirner J. 6-Sulfo LacNAc, a Novel Carbohydrate Modification of PSGL-1, Defines an Inflammatory Type of Human Dendritic Cells. Immunity (2002) 17(3):289–301. doi: 10.1016/s1074-7613(02)00393-x 12354382

[31] Gutiérrez-Bautista JF Rodriguez-Nicolas A Rosales-Castillo A Jiménez P Garrido F Anderson P. Negative Clinical Evolution in COVID-19 Patients Is Frequently Accompanied With an Increased Proportion of Undifferentiated Th Cells and a Strong Underrepresentation of the Th1 Subset. Front Immunol (2020) 11:596553. doi: 10.3389/fimmu.2020.596553 33324414 PMC7726249

[32] de Campos-Mata L Tejedor Vaquero S Tachó-Piñot R Piñero J Grasset EK Arrieta Aldea I. SARS-CoV-2 Sculpts the Immune System to Induce Sustained Virus-Specific Naïve-Like and Memory B-Cell Responses. Clin Transl Immunol (2021) 10(9):e1339. doi: 10.1002/cti2.1339 PMC8418925 34504693

[33] Su CM Wang L Yoo D. Activation of NF-κb and Induction of Proinflammatory Cytokine Expressions Mediated by ORF7a Protein of SARS-CoV-2. Sci Rep (2021) 11(1):13464. doi: 10.1038/s41598-021-92941-2 34188167 PMC8242070

[34] Khalil BA Elemam NM Maghazachi AA. Chemokines and Chemokine Receptors During COVID-19 Infection. Comput Struct Biotechnol J (2021) 19:976–88. doi: 10.1016/j.csbj.2021.01.034 PMC7859556 33558827

[35] Xu ZS Shu T Kang L Wu D Zhou X Liao BW. Temporal Profiling of Plasma Cytokines, Chemokines and Growth Factors From Mild, Severe and Fatal COVID-19 Patients. Signal Transduct Target Ther (2020) 5(1):100. doi: 10.1038/s41392-020-0211-1 32561706 PMC7303571

[36] Bouadma L Wiedemann A Patrier J Surénaud M Wicky PH Foucat E. Immune Alterations in a Patient With SARS-CoV-2-Related Acute Respiratory Distress Syndrome. J Clin Immunol (2020) 40(8):1082–92. doi: 10.1007/s10875-020-00839-x PMC7443154 32829467

[37] Kong SL Chui P Lim B Salto-Tellez M. Elucidating the Molecular Physiopathology of Acute Respiratory Distress Syndrome in Severe Acute Respiratory Syndrome Patients. Virus Res (2009) 145(2):260–9. doi: 10.1016/j.virusres.2009.07.014 PMC7114434 19635508

[38] Yan Y Jiang X Wang X Liu B Ding H Jiang M. CCL28 Mucosal Expression in SARS-CoV-2-Infected Patients With Diarrhea in Relation to Disease Severity. J Infect (2021) 82(1):e19–21. doi: 10.1016/j.jinf.2020.08.042 PMC7833095 32871180

[39] Wollina U Karadağ AS Rowland-Payne C Chiriac A Lotti T. Cutaneous Signs in COVID-19 Patients: A Review. Dermatol Ther (2020) 33(5):e13549. doi: 10.1111/dth.13549 32390279 PMC7273098

[40] Menezes SM Decanine D Brassat D Khouri R Schnitman SV Kruschewsky R. CD80+ and CD86+ B Cells as Biomarkers and Possible Therapeutic Targets in HTLV-1 Associated Myelopathy/Tropical Spastic Paraparesis and Multiple Sclerosis. J Neuroinflamm (2014) 11:18. doi: 10.1186/1742-2094-11-18 PMC3922160 24472094

[41] Huang Y Wei B Gao X Deng Y Wu W. Expression of CD80 and CD86 on B Cells During Coxsackievirus B3-Induced Acute Myocarditis. Cent Eur J Immunol (2019) 44(4):364–9. doi: 10.5114/ceji.2019.92786 PMC7050056 32140047

[42] Suvas S Singh V Sahdev S Vohra H Agrewala JN. Distinct Role of CD80 and CD86 in the Regulation of the Activation of B Cell and B Cell Lymphoma. J Biol Chem (2002) 277(10):7766–75. doi: 10.1074/jbc.M105902200 11726649

[43] Wang JH Wu Q Yang P Li H Li J Mountz JD. Type I Interferon-Dependent CD86(high) Marginal Zone Precursor B Cells Are Potent T Cell Costimulators in Mice. Arthritis Rheum (2011) 63(4):1054–64. doi: 10.1002/art.30231 PMC3310977 21225691

[44] Wildner NH Ahmadi P Schulte S Brauneck F Kohsar M Lütgehetmann M. B Cell Analysis in SARS-CoV-2 Versus Malaria: Increased Frequencies of Plasmablasts and Atypical Memory B Cells in COVID-19. J Leukoc Biol (2021) 109(1):77–90. doi: 10.1002/JLB.5COVA0620-370RR 33617048 PMC10016889

[45] Thibult ML Mamessier E Gertner-Dardenne J Pastor S Just-Landi S Xerri L. PD-1 is a Novel Regulator of Human B-Cell Activation. Int Immunol (2013) 25(2):129–37. doi: 10.1093/intimm/dxs098 23087177

[46] Beaudoin-Bussières G Laumaea A Anand SP Prévost J Gasser R Goyette G. Decline of Humoral Responses Against SARS-CoV-2 Spike in Convalescent Individuals. mBio (2020) 11(5):e02590–20. doi: 10.1128/mBio.02590-20 PMC7569150 33067385

[47] Long QX Liu BZ Deng HJ Wu GC Deng K Chen YK. Antibody Responses to SARS-CoV-2 in Patients With COVID-19. Nat Med (2020) 26(6):845–8. doi: 10.1038/s41591-020-0897-1 32350462

[48] Ogega CO Skinner NE Blair PW Park HS Littlefield K Ganesan A. Durable SARS-CoV-2 B Cell Immunity After Mild or Severe Disease. medRxiv (2020) 10.28.20220996. doi: 10.1101/2020.10.28.20220996 PMC8011891 33571162

[49] Denton AE Linterman MA. Stromal Networking: Cellular Connections in the Germinal Centre. Curr Opin Immunol (2017) 45:103–11. doi: 10.1016/j.coi.2017.03.001 28319729

[50] Oropallo MA Cerutti A. Germinal Center Reaction: Antigen Affinity and Presentation Explain it All. Trends Immunol (2014) 35(7):287–9. doi: 10.1016/j.it.2014.06.001 PMC4174395 24934509

[51] Li X Zhang Q Zhang W Ye G Ma Y Wen C. Expanded Circulating Follicular Dendritic Cells Facilitate Immune Responses in Chronic HBV Infection. J Transl Med (2020) 18(1):417. doi: 10.1186/s12967-020-02584-6 33160362 PMC7648402

[52] Good-Jacobson KL Szumilas CG Chen L Sharpe AH Tomayko MM Shlomchik MJ. PD-1 Regulates Germinal Center B Cell Survival and the Formation and Affinity of Long-Lived Plasma Cells. Nat Immunol (2010) 11(6):535–42. doi: 10.1038/ni.1877 PMC2874069 20453843

[53] Juno JA Tan HX Lee WS Reynaldi A Kelly HG Wragg K. Humoral and Circulating Follicular Helper T Cell Responses in Recovered Patients With COVID-19. Nat Med (2020) 26(9):1428–34. doi: 10.1038/s41591-020-0995-0 32661393

[54] Gong F Dai Y Zheng T Cheng L Zhao D Wang H. Peripheral CD4+ T Cell Subsets and Antibody Response in COVID-19 Convalescent Individuals. J Clin Invest (2020) 130(12):6588–99. doi: 10.1172/JCI141054 PMC7685722 32841212

[55] Ni L Ye F Cheng ML Feng Y Deng YQ Zhao H. Detection of SARS-CoV-2-Specific Humoral and Cellular Immunity in COVID-19 Convalescent Individuals. Immunity (2020) 52(6):971–7.e3. doi: 10.1016/j.immuni.2020.04.023 32413330 PMC7196424

[56] Long Y Zhao X Liu C Xia C. Activated Inducible Co-Stimulator-Positive Programmed Cell Death 1-Positive Follicular Helper T Cells Indicate Disease Activity and Severity in Ulcerative Colitis Patients. Clin Exp Immunol (2020) 202(1):106–18. doi: 10.1111/cei.13485 PMC7488121 32621310

[57] Fan X Jin T Zhao S Liu C Han J Jiang X. Circulating CCR7+ICOS+ Memory T Follicular Helper Cells in Patients With Multiple Sclerosis. PloS One (2015) 10(7):e0134523. doi: 10.1371/journal.pone.0134523 26231034 PMC4521720

[58] Kim JW Lee J Hong SM Cho ML Park SH. Circulating CCR7loPD-1hi Follicular Helper T Cells Indicate Disease Activity and Glandular Inflammation in Patients With Primary Sjögren’s Syndrome. Immune Netw (2019) 19(4):e26. doi: 10.4110/in.2019.19.e26 31501714 PMC6722269

[59] Zhang J Wu Q Liu Z Wang Q Wu J Hu Y. Spike-Specific Circulating T Follicular Helper Cell and Cross-Neutralizing Antibody Responses in COVID-19-Convalescent Individuals. Nat Microbiol (2021) 6(1):51–8. doi: 10.1038/s41564-020-00824-5 33199863

[60] De Giorgi V West KA Henning AN Chen LN Holbrook MR Gross R. Naturally Acquired SARS-CoV-2 Immunity Persists for Up to 11 Months Following Infection. J Infect Dis (2021) 224(8):1294–304. doi: 10.1093/infdis/jiab295 PMC8195007 34089610

[61] Glöckner S Hornung F Baier M Weis S Pletz MW Deinhardt-Emmer S. Robust Neutralizing Antibody Levels Detected After Either SARS-CoV-2 Vaccination or One Year After Infection. Viruses (2021) 13(10):2003. doi: 10.3390/v13102003 34696428 PMC8537517

[62] Sakharkar M Rappazzo CG Wieland-Alter WF Hsieh CL Wrapp D Esterman ES. Prolonged Evolution of the Human B Cell Response to SARS-CoV-2 Infection. Sci Immunol (2021) 6(56):eabg6916. doi: 10.1126/sciimmunol.abg6916 33622975 PMC8128290

[63] Kaneko N Kuo HH Boucau J Farmer JR Allard-Chamard H Mahajan VS. Loss of Bcl-6-Expressing T Follicular Helper Cells and Germinal Centers in COVID-19. Cell (2020) 183(1):143–57.e13. doi: 10.1016/j.cell.2020.08.025 32877699 PMC7437499

[64] Duan YQ Xia MH Ren L Zhang YF Ao QL Xu SP. Deficiency of Tfh Cells and Germinal Center in Deceased COVID-19 Patients. Curr Med Sci (2020) 40(4):618–24. doi: 10.1007/s11596-020-2225-x PMC7412778 32767259

[65] Gallais F Gantner P Bruel T Velay A Planas D Wendling MJ. Evolution of Antibody Responses Up to 13 Months After SARS-CoV-2 Infection and Risk of Reinfection. EBioMedicine (2021) 71:103561. doi: 10.1016/j.ebiom.2021.103561 34455390 PMC8390300

[66] Dobaño C Ramírez-Morros A Alonso S Vidal-Alaball J Ruiz-Olalla G Vidal M. Persistence and Baseline Determinants of Seropositivity and Reinfection Rates in Health Care Workers Up to 12.5 Months After COVID-19. BMC Med (2021) 19(1):155. doi: 10.1186/s12916-021-02032-2 34183003 PMC8237770

