# Supplementary material for: Comprehensive Flow Cytometry Profiling of the Immune System in COVID-19 Convalescent Individuals
Source: Front Immunol. 2022 Jan 6;12:793142. doi: 10.3389/fimmu.2021.793142 (PMC8771913; doi:10.3389/fimmu.2021.793142)
Supplement: Supplementary file 1 [file DataSheet_1.pdf]

Supplementary Material

Supplemental S1

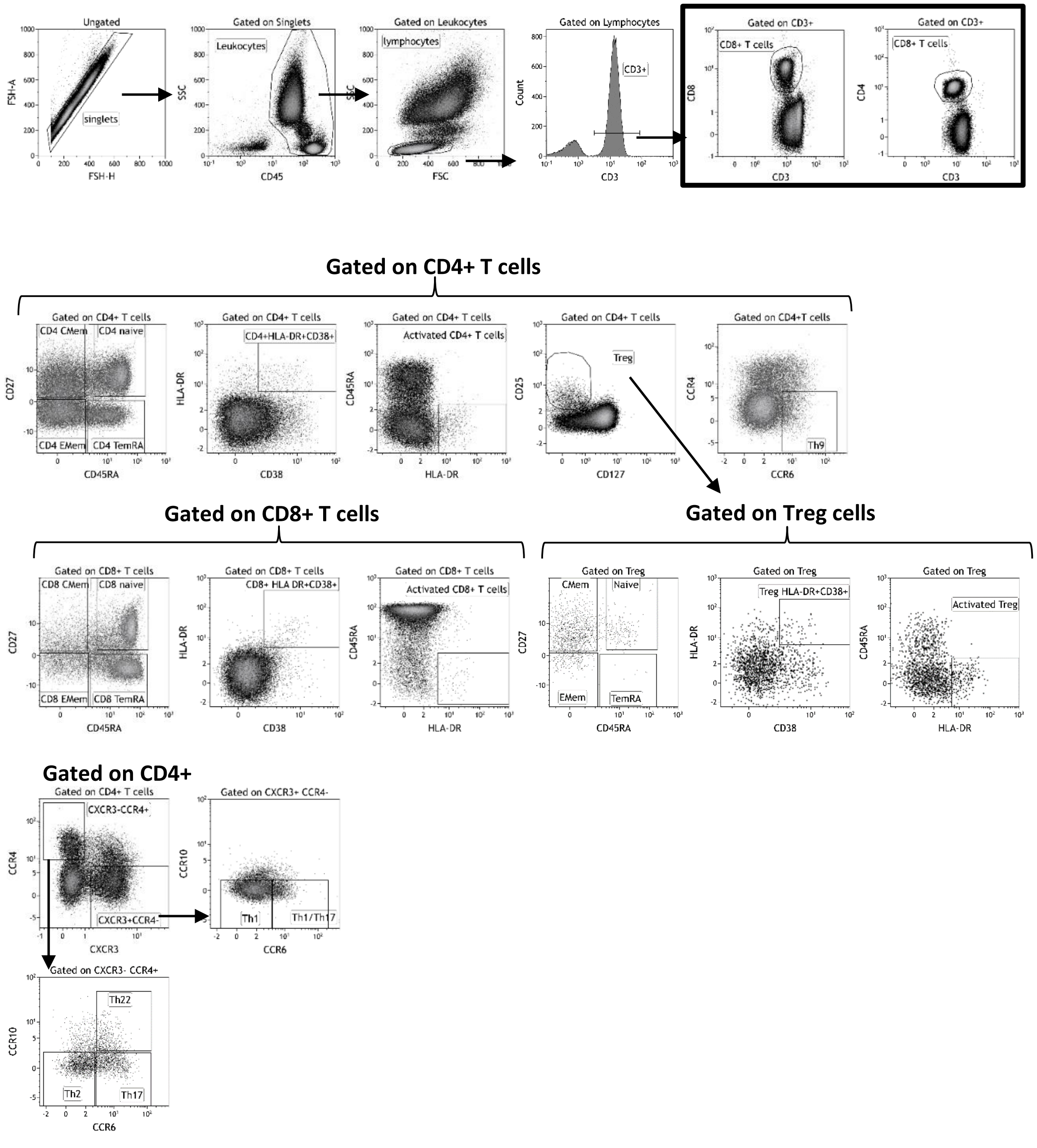

Figure S1. Traditional manual gating strategy for the analysis of the T-cell panel. Gating strategies to define T-cell subsets used in COV and CT-group individuals. Representative examples of flow cytometry plots determined on whole blood labeled from one individual.

Supplemental S2

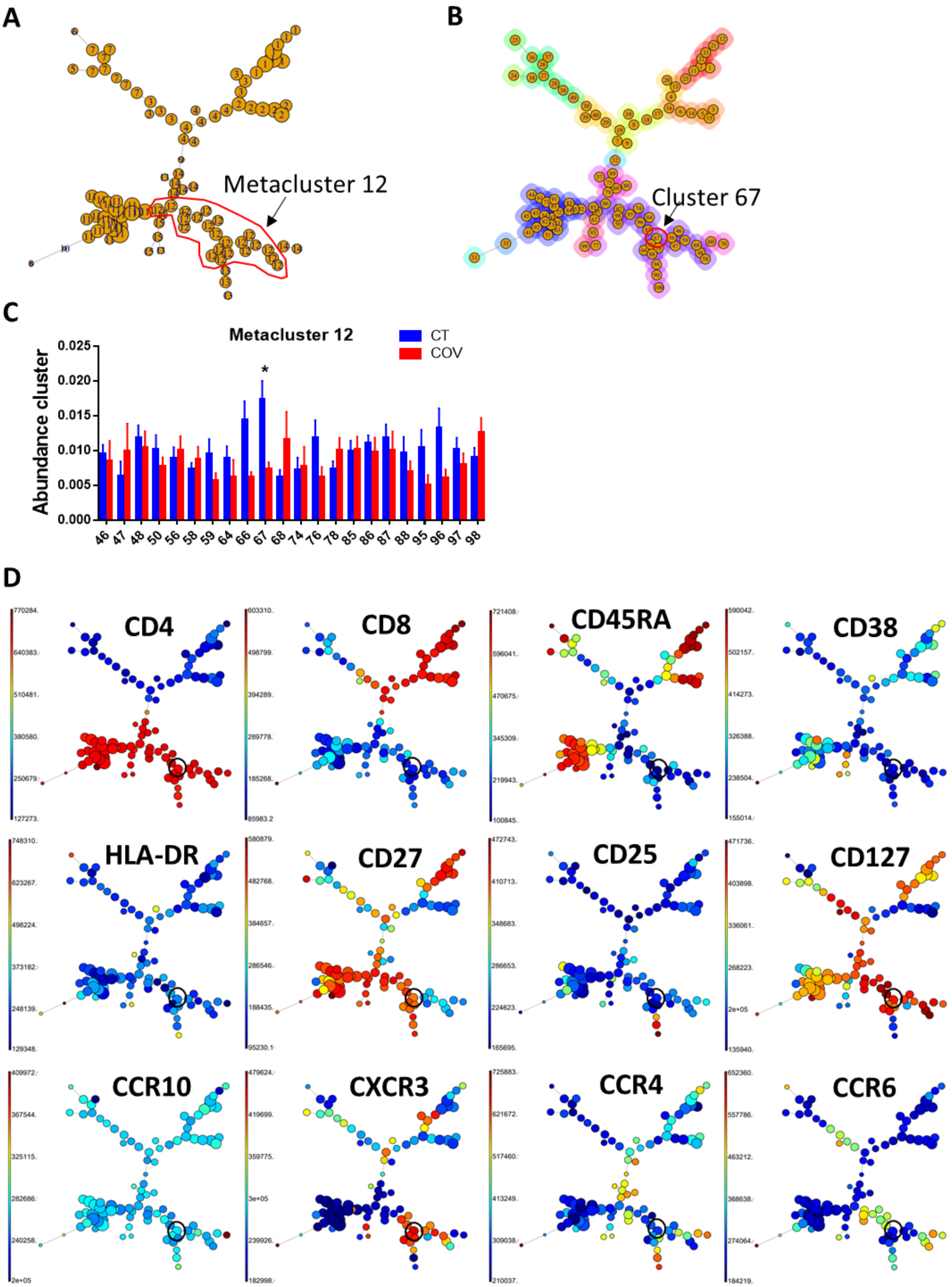

Figure S2. FlowSOM High-dimensional analysis of T cells cytometry panel subsets. (**A**) Tree graphical representation for the metaclusters obtained by FlowSOM analysis; metacluster 12 is bounded with a red line. (**B**) Tree graphical representation of the 15 metaclusters obtained by FlowSOM analysis. Each metacluster is in a different color. Cluster 67, the only cluster significantly different between groups in metacluster 12, is bounded with a red circle. (**C**) The abundance of clusters was obtained through FlowSOM analysis. Two-way ANOVA with Benjamini–Hochberg adjustment for multiple testing. Median ± SEM. *$p < 0.05$. (**D**) Tree graphical representation shows all the markers' expressions (CD4, CD8, CD45RA. CD38, HLA-DR, CD27, CD25, CD127, CCR10, CXCR3 CCR4, and CCR6) of the T-cell panel after FlowSOM analysis. According to this graphic, cluster 67 was CD4+ CD45RAneg CCR4neg CCR10neg CD27+ CCR6neg CXCR3+ CD127+. The color scale (left axis) represents the intensity of the marker's expression, with dark red for high-intensity expression and dark blue for no expression.

Supplemental S3

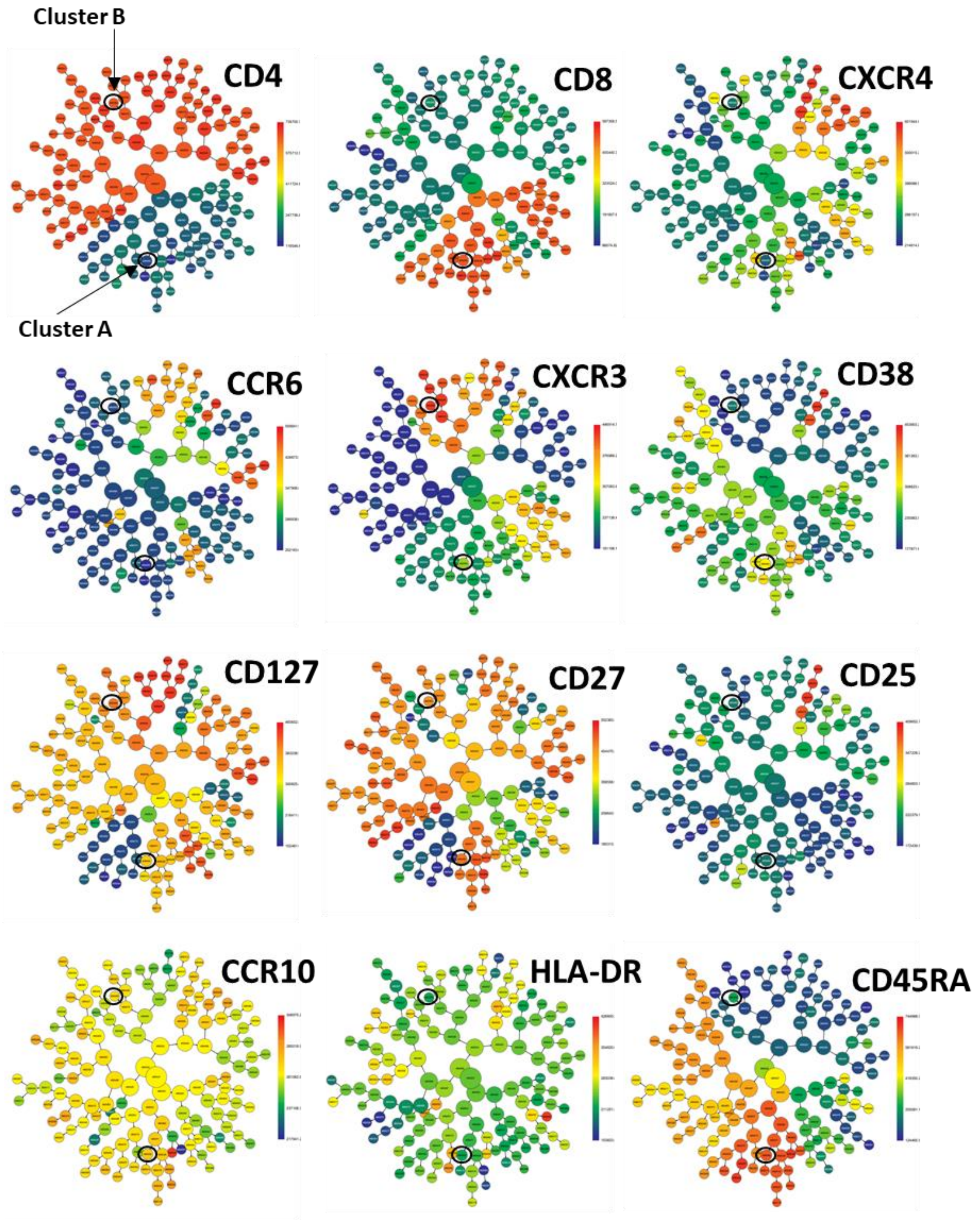

Figure S3. Graphics from CITRUS analysis of the T cell cytometry panel. Clusters A and B, depicted by black circles, were more abundant in the CT group than in the COV group. The tree graphical representation shows all the markers' expression (CD4, CD8, CD45RA. CD38, HLA-DR, CD27, CD25, CD127, CCR10, CXCR3, CCR4, and CCR6) of the T-cell panel. The color scale (right axis) represents the intensity of the marker expression, indicating positive expression in dark red and negative expression in dark blue. Clusters A and B are bounded with black circles. Cluster A expressed CD8+ CD38+ CD127+ CD27+ CCR10+ and CD45RA+ and cluster B expressed CD4+ CXCR3+ CD127+ CD27+ and CCR10+.

Supplemental S4

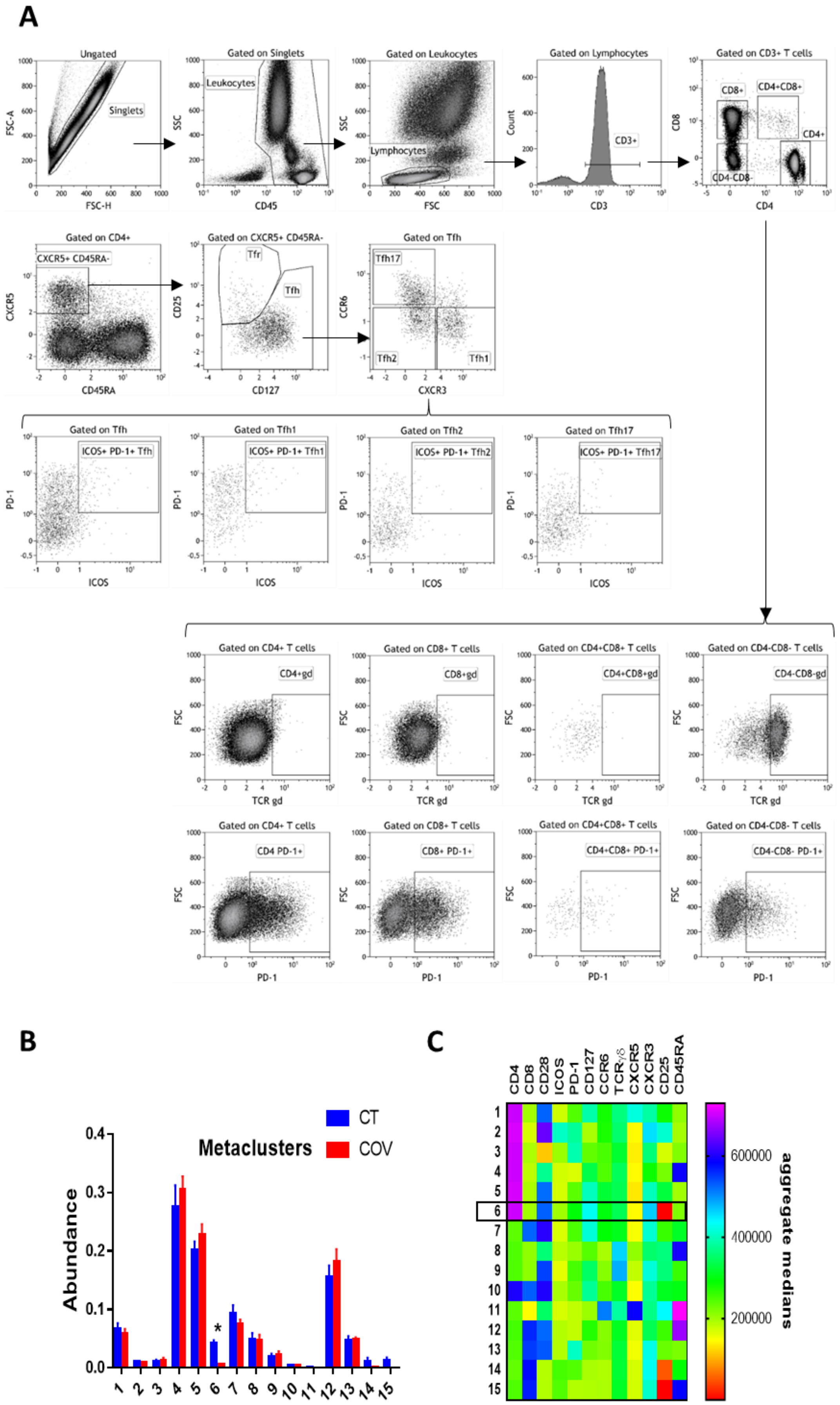

Figure S4. Manual gating strategy and high-dimensional analysis of the Tfh–Tγδ cell cytometry panel. (**A**) Gating strategies to define T-cell subsets used in COV and CT-group individuals. Representative examples of flow cytometry plots determined on whole blood labeled from one individual. (**B**) The abundance of metaclusters was obtained through FlowSOM analysis. Median ± SEM, *$p < 0.05$. (**C**) Heat map from FlowSOM analysis of the aggregate medians for all the markers' intensity expression into the Tfh–Tγδ cell cytometry panel. On the Y-axis (left) are the 15 metaclusters' numbers, identified by FlowSOM, and on the X-axis (top) are all the studied markers. Red and purple indicate the minimum and maximum median intensity expression on the color scale on the right Y-axis, respectively. Metacluster 6, bounded with the black line, was the only metacluster identified as having a significantly greater abundance in the CT than in the COV group by two-way ANOVA with Benjamini–Hochberg adjustment for multiple testing

Supplemental S5

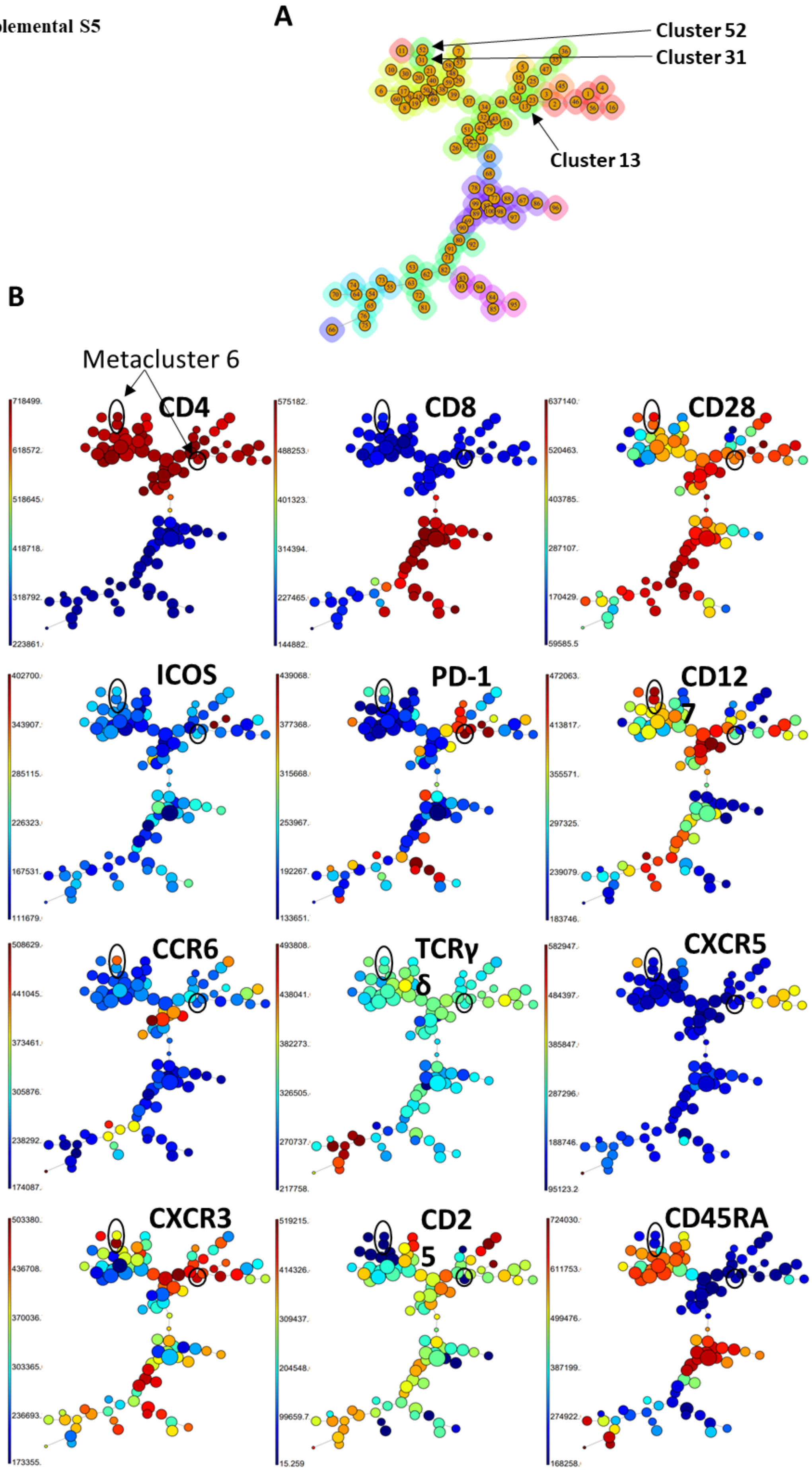

Figure S5. Tree graphical representation of the FlowSOM analysis of the Tfh–Tγδ cell cytometry panel. (**A**) Tree graphical representation of the 15 metaclusters detected by FlowSOM analysis, with each metacluster in a different color. Clusters 13, 31, and 52 were identified in metacluster 6 and were more abundant in the CT group than in the COV group. (**B**) Tree graphical representation showing the expression of all the markers of the Tfh–Tγδ cell panel (CD4, CD8, CD28, ICOS, PD-1, CD127, CCR6, TCRγδ, CXCR5, CXCR3, CD25, and CD45RA), obtained by FlowSOM analysis. Clusters 13, 31 and 52 are bounded with black circles. The color scale (left axis) represents the intensity of the marker's expression, with dark red for high expression and dark blue for no expression.

Supplemental S6

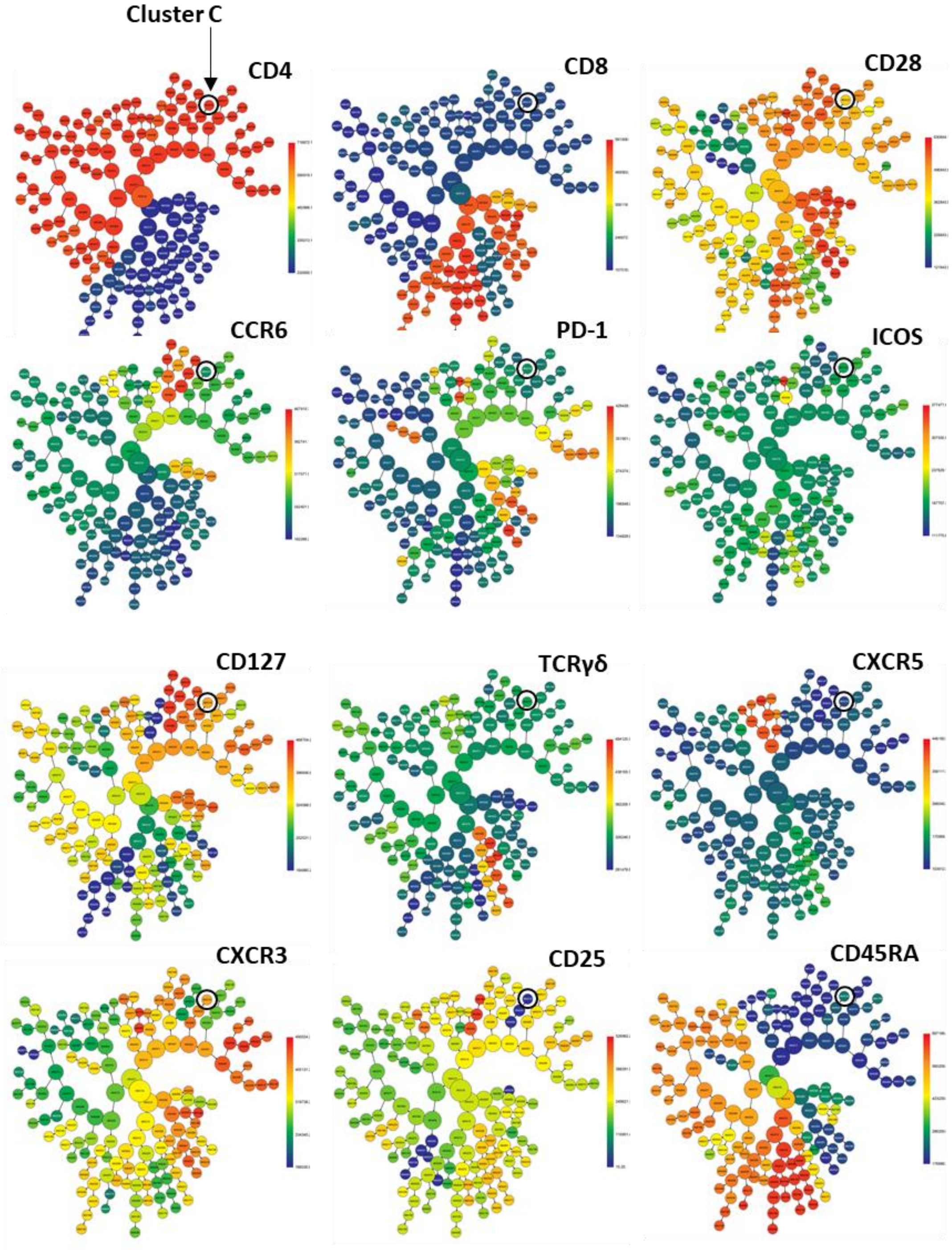

Figure S6. Graphics from CITRUS analysis of the Tfh–Tγδ cell cytometry panel. Cluster C, depicted by a black circle, was significantly more abundant in the CT group than in the COV group, as determined by CITRUS analysis. Tree graphical representation showing the expression of all the markers of the Tfh- Tγδ-cell panel (CD4, CD8, CD28, ICOS, PD-1, CD127, CCR6, TCRγδ, CXCR5, CXCR3, CD25, and CD45RA). The color scale (right axis) represents the intensity of the marker's expression, with dark red for high expression and dark blue for no expression. Cluster C was determined to be CD4+ CD28+ CD127+ CXCR3+.

Supplemental S7

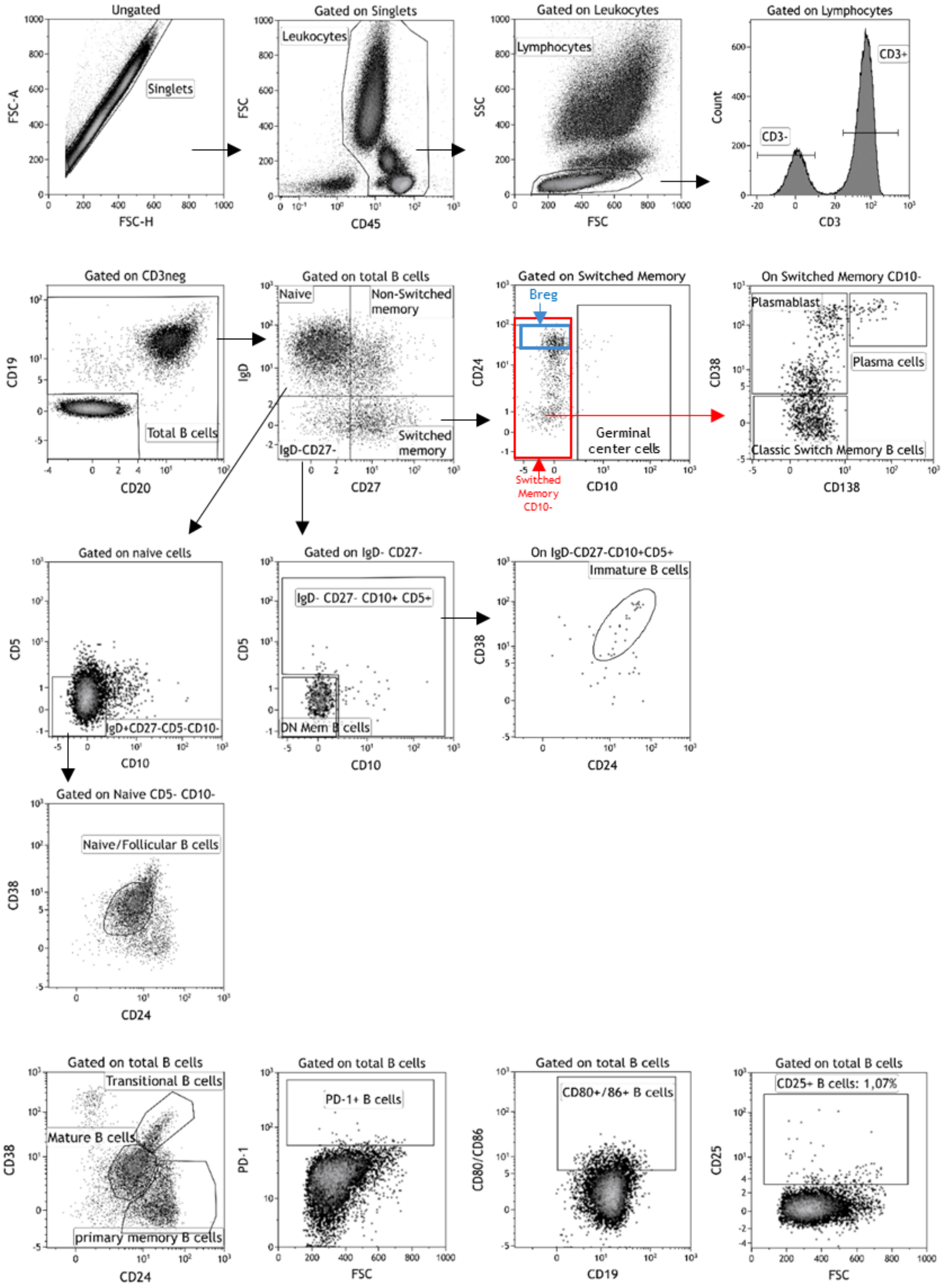

Figure S7. Traditional manual gating strategy for the analysis of the B-cell panel. Gating strategies to define B-cell subsets used in COV and CT group individuals. Representative examples of flow cytometry plots determined on whole blood labeled from one individual. The blue rectangle defines the Breg population, and the red rectangle the Switched memory CD10neg.

Supplemental S8

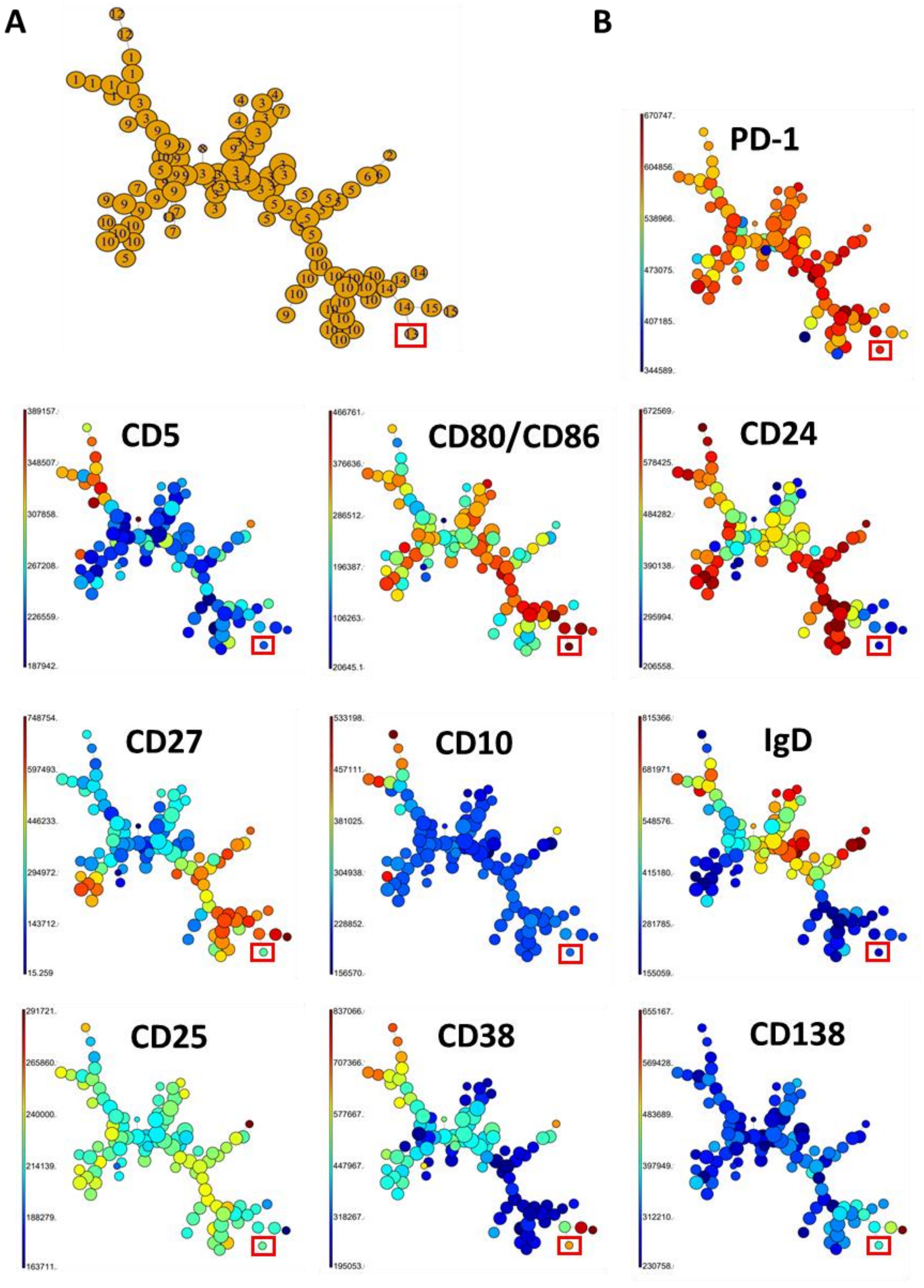

Figure S8. High-dimensional analysis of the B cell cytometry panel. (**A**) Tree graphical representation for the 15 metaclusters obtained by the FlowSOM analysis. Metacluster 13, depicted with a red square, was determined by a FlowSOM analysis as being significantly different between the CT and COV groups. (**B**) Tree graphical representation showing the expression of all the markers of the B-cell panel (PD-1, CD5, CD80/CD86, CD24, CD27, CD10, IgD, CD25, CD38, and CD138). The color scale (left axis) represents the intensity of the marker's expression, with dark red for high expression and dark blue for no expression. The red square indicates metacluster 13, expressing CD19+ CD20+ CD80/CD86+ CD38+.

Supplemental S9

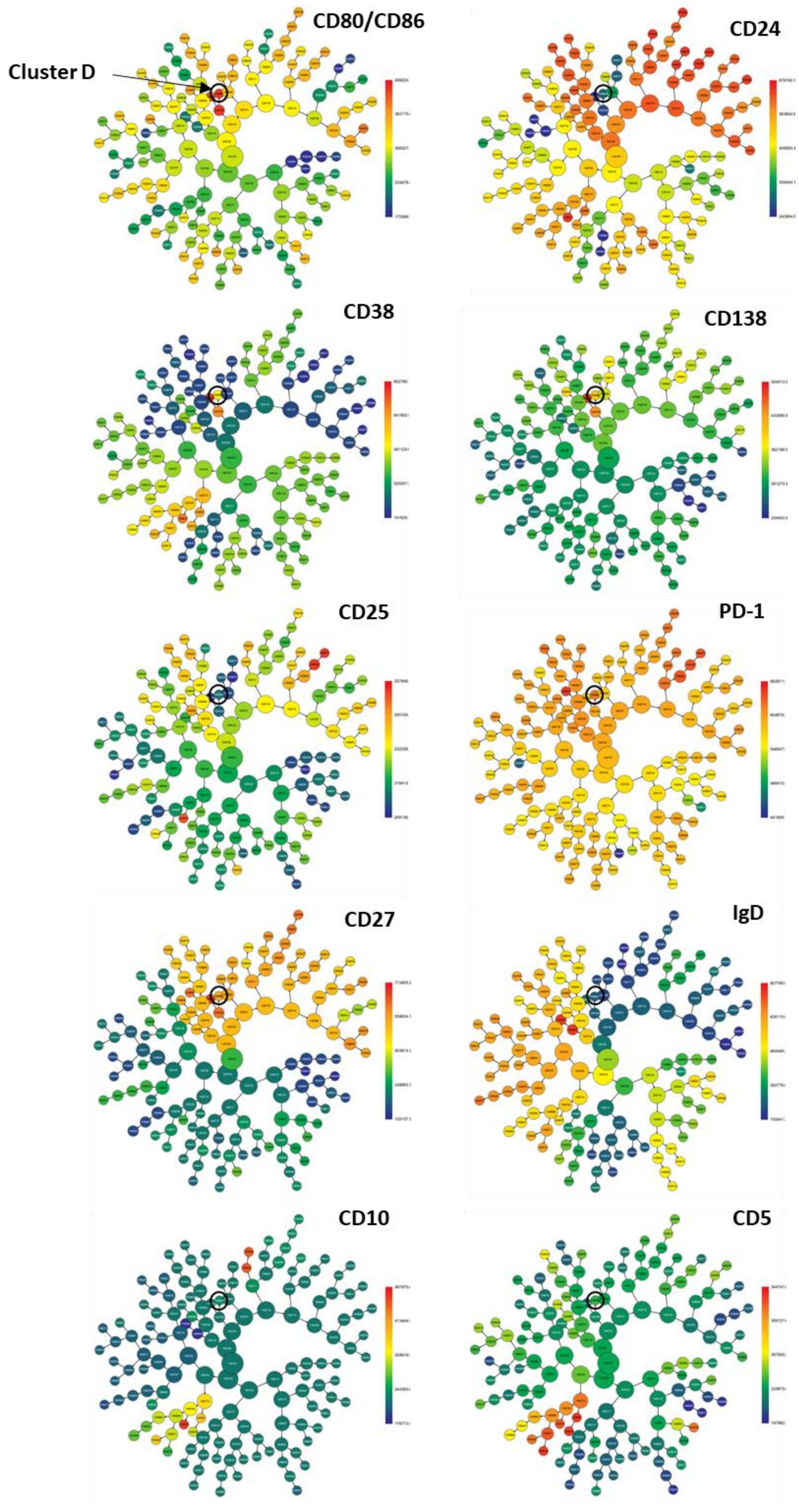

Figure S9. Graphics from CITRUS analysis of the B-cell cytometry panel. Tree graphical representation of clusters determined by CITRUS analysis. Cluster D, depicted as a black circle, was more abundant in the COV group than in the CT group. Markers' intensities are depicted by a color scale (right axis), with dark red for high expression and dark blue for no expression. We identified that cluster D was defined as CD80/CD86+ CD27+ CD38+ CD138+ PD-1+.

Supplemental S10

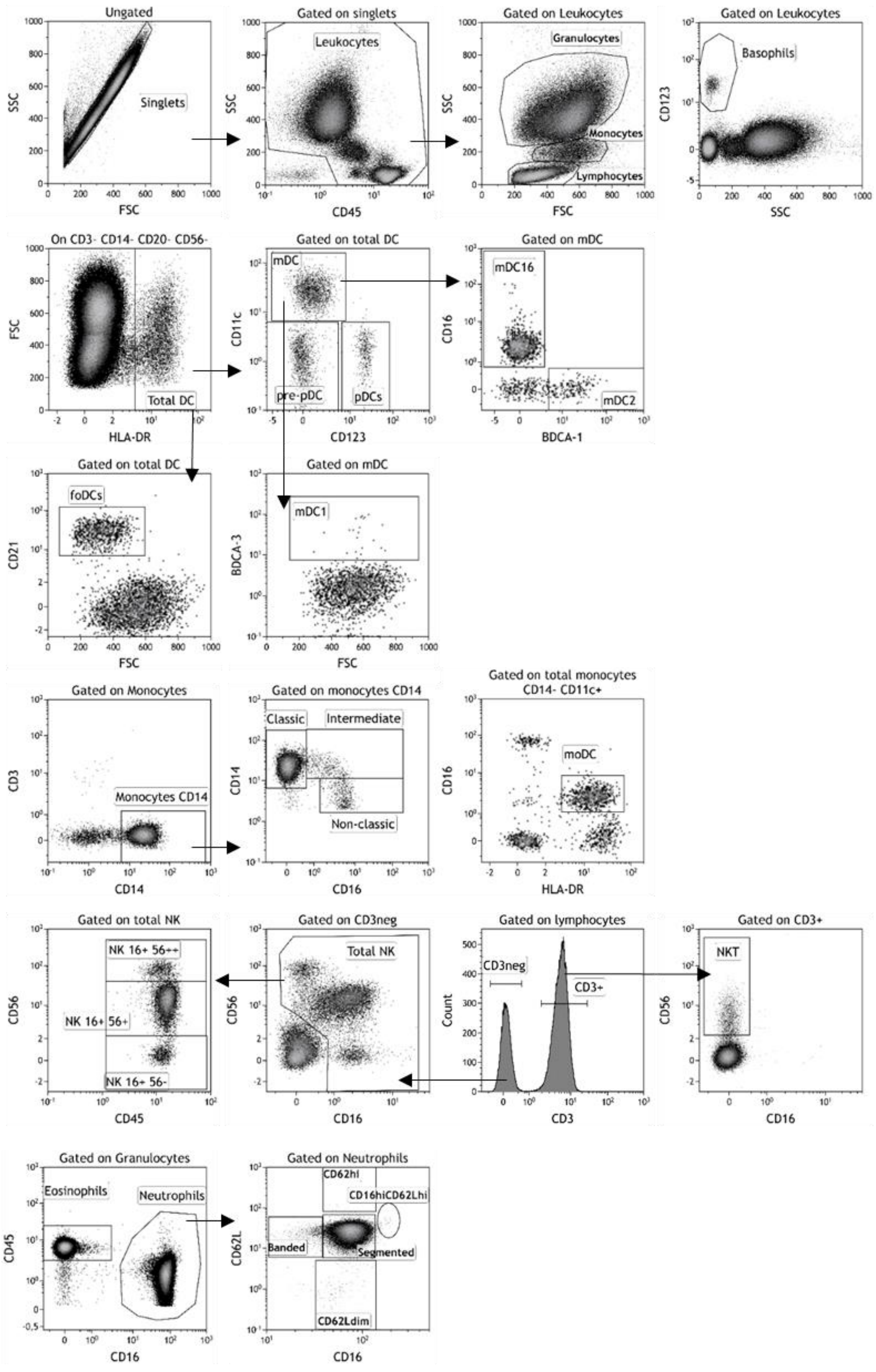

Figure S10. Traditional manual gating strategy for the analysis of the innate immune cells' panel. Gating strategies to define innate immune cells' subsets used in COV and CT group individuals. Representative examples of flow cytometry plots determined on whole blood labeled from one individual.

Supplemental S11

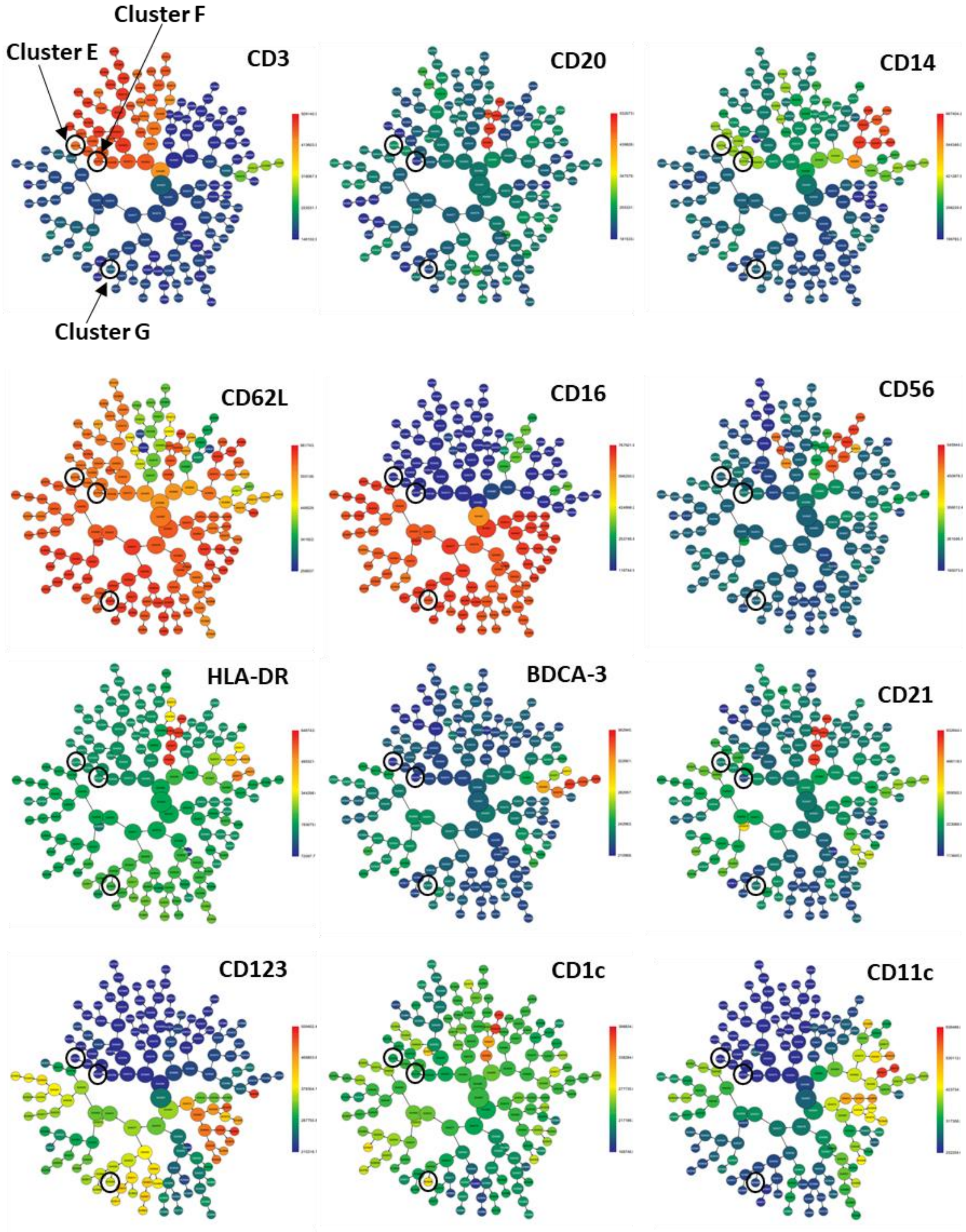

Figure S11. Graphics from CITRUS analysis of the innate cell cytometry panel. Tree graphical representation of clusters determined by CITRUS analysis. Clusters E, F, and G, depicted by black circles, were more abundant in the COV group than in the CT group. Markers' intensities for CD62L, CD16, CD56, HLA-DR, BDCA-3, CD21, CD123, CD1c, and CD11c are depicted by a color scale (right axis), with dark red for high expression and dark blue for no expression. We identified that clusters E and F expressed almost the same markers, CD14+ CD3+ CD62L+. Cluster G was HLA-DRneg CD11cneg CD62L+ CD16+ CD123+ CD1cint.

Table S1

Antibody	Fluorescence	Panel
BDCA-3	FITC	Innate cells
CCR10	APC	B-cells
CCR4	BV605	T-cells
CCR6	PE-Vio615	T-cells
		Tfh-Tγδ cells
CD10	PE-Vio770	B-cells
CD11c	APC5	Innate cells
CD123	APC-Vio770	Innate cells
CD127	PE	T-cells
		Tfh-Tγδ cells
CD138	APC	B-cells
CD14	Vioblue	Innate cells
CD16	PE-Vio770	Innate cells
CD19	PE-Vio615	B-cells
CD1c	APC	Innate cells
CD20	PerCP	B-cells
		Innate cells
CD21	PE	Innate cells
CD24	VioGreen	B-cells
CD25	PE-Vio770	T-cells
	APC-Fire750	B-cells
	APC-R700	Tfh-Tγδ cells
CD27	PerCP-Vio700	T-cells
CD28	BV570	Tfh-Tγδ cells
CD3	VioGreen	T-cells
	VioGreen	Tfh-Tγδ cells
	BV605	B-cells
	VioGreen	Innate cells
CD38	BV650	T-cells
	APC	B-cells
CD4	VioBlue	T-cells
	VioBlue	Tfh-Tγδ cells
CD45	PE-Cy5	T-cells
	PE-Cy5	Tfh-Tγδ cells
	BV570	B-cells
	BV570	Innate cells
CD45RA	APC-Vio770	B-cells
		Tfh-Tγδ cells
CD5	FITC	B-cells
CD56	PE-Vio615	Innate cells
CD62L	BV650	Innate cells
CD80	BV650	B-cells
CD86	BV650	B-cells
CD8a	BV570	T-cells
		Tfh-Tγδ cells
CXCR3	VioBright	T-cells
	APC	Tfh-Tγδ cells
	PE-Vio770	Tfh-Tγδ cells
HLA-DR	Alexa Fluor 700	B-cells
	PerCP-Vio700	NK-DC
ICOS	BV605	Tfh-Tγδ cells
IgD	PE	B-cells
PD-1	VioBright 515	Tfh-Tγδ cells
	BV421	B-cells
TCR-γδ	PerCP-Vio700	Tfh-Tγδ cells

Table S1: Antibodies used in the four cytometry panels.
For each antibody, the fluorochrome and panel where it was used is indicated.
